# A Genome-wide Association Study Identifies *SERPINB10, CRLF3, STX7*, *LAMP3, IFNG-AS1*, and *KRT80* As Risk Loci Contributing to Cutaneous Leishmaniasis in Brazil

**DOI:** 10.1093/cid/ciaa1230

**Published:** 2020-08-23

**Authors:** Léa C Castellucci, Lucas Almeida, Svetlana Cherlin, Michaela Fakiola, Richard W Francis, Edgar M Carvalho, Anadílton Santos da Hora, Tainã Souza do Lago, Amanda B Figueiredo, Clara M Cavalcanti, Natalia S Alves, Katia L P Morais, Andréa Teixeira-Carvalho, Walderez O Dutra, Kenneth J Gollob, Heather J Cordell, Jenefer M Blackwell

**Affiliations:** 1 National Institute of Science and Technology in Tropical Diseases, Brazil; 2 Federal University of Bahia, Salvador, Brazil; 3 Population Health Sciences Institute, Newcastle University, Newcastle-Upon-Tyne, United Kingdom; 4 National Institute of Molecular Genetics “Romeo ed Enrica Invernizzi,” Milan, Italy; 5 Telethon Kids Institute, University of Western Australia, Nedlands, Australia; 6 International Center for Research, AC Camargo Cancer Center, São Paulo, Brazil; 7 Instituto Rene Rachou of Fundação Oswaldo Cruz (FIOCRUZ-Minas), Belo Horizonte, Brazil; 8 Instituto de Ciências Biológicas, Universidade Federal de Minas Gerais, Belo Horizonte, Brazil; 9 Núcleo de Ensino e Pesquisa, Instituto Mario Penna, Belo Horizonte, Brazil; 10 Department of Pathology, University of Cambridge, Cambridge, United Kingdom

**Keywords:** *Leishmania*, GWAS, post-GWAS integrated analysis, interferon-γ, *IFNG-AS1*

## Abstract

**Background:**

Our goal was to identify genetic risk factors for cutaneous leishmaniasis (CL) caused by *Leishmania braziliensis*.

**Methods:**

Genotyping 2066 CL cases and 2046 controls using Illumina HumanCoreExomeBeadChips provided data for 4 498 586 imputed single-nucleotide variants (SNVs). A genome-wide association study (GWAS) using linear mixed models took account of genetic diversity/ethnicity/admixture. Post-GWAS positional, expression quantitative trait locus (eQTL) and chromatin interaction mapping was performed in Functional Mapping and Annotation (FUMA). Transcriptional data were compared between lesions and normal skin, and cytokines measured using flow cytometry and Bioplex assay.

**Results:**

Positional mapping identified 32 genomic loci associated with CL, none achieving genome-wide significance (*P* < 5 × 10^−8^). Lead SNVs at 23 loci occurred at protein coding or noncoding RNA genes, 15 with eQTLs for functionally relevant cells/tissues and/or showing differential expression in lesions. Of these, the 6 most plausible genetic risk loci were *SERPINB10* (*P*_imputed_1000G_ = 2.67 × 10^−6^), *CRLF3* (*P*_imputed_1000G_ = 5.12 × 10^−6^), *STX7* (*P*_imputed_1000G_ = 6.06 × 10^−6^), *KRT80* (*P*_imputed_1000G_ = 6.58 × 10^−6^), *LAMP3* (*P*_imputed_1000G_ = 6.54 × 10^−6^), and *IFNG-AS1* (*P*_imputed_1000G_ = 1.32 × 10^−5^). *LAMP3* (*P*_adjusted_ = 9.25 × 10^−12^; +6-fold), *STX7* (*P*_adjusted_ = 7.62 × 10^−3^; +1.3-fold), and *CRLF3* (*P*_adjusted_ = 9.19 × 10^−9^; +1.97-fold) were expressed more highly in CL biopsies compared to normal skin; *KRT80* (*P*_adjusted_ = 3.07 × 10^−8^; −3-fold) was lower. Multiple cis-eQTLs across *SERPINB10* mapped to chromatin interaction regions of transcriptional/enhancer activity in neutrophils, monocytes, B cells, and hematopoietic stem cells. Those at *IFNG-AS1* mapped to transcriptional/enhancer regions in T, natural killer, and B cells. The percentage of peripheral blood CD3^+^ T cells making antigen-specific interferon-γ differed significantly by *IFNG-AS1* genotype.

**Conclusions:**

This first GWAS for CL identified multiple genetic risk loci including a novel lead to understanding CL pathogenesis through regulation of interferon-γ by IFNG antisense RNA 1.

## Teaser

Genome-wide analysis of 2066 cases and 2046 controls, together with genotypic differences in antigen-specific interferon-γ made by CD3^+^ T cells, identifies *IFNG-AS1* as a genetic risk factor for cutaneous leishmaniasis caused by *Leishmania braziliensis*.

American cutaneous leishmaniasis (ACL) caused by *Leishmania braziliensis* has multiple presentations including cutaneous (CL), mucosal (ML), and disseminated (DL) leishmaniasis. ML and DL are generally preceded by CL. The common CL form of disease is associated with localized skin lesions, mainly ulcers, on exposed body parts. While normally self-limiting, the degree of pathology and rate of healing varies, with lesions leaving lifelong scars. Not all infected individuals go on to develop disease. Subclinical infection is associated with *Leishmania*-specific cellular immune responses, measured as delayed-type hypersensitivity (DTH) skin test responses [1]. *Leishmania* antigen-stimulated peripheral blood lymphocytes also produce interferon gamma (IFN-γ) and tumor necrosis factor (TNF) in subclinical infection, but at lower levels than CL [1]. In a longitudinal study, IFN-γ was associated with protection, but a positive skin-test response was not [2]. Indeed, a positive DTH response has high sensitivity for diagnosis of *L. braziliensis* CL [1, 3]. All forms of ACL are associated with exaggerated cellular immunity. In CL, there is a positive correlation between the frequency of CD4^+^ T cells expressing IFN-γ and TNF and lesion size [4], with higher levels in ML than CL [5]. The outcome of *L. braziliensis* infection is determined by a fine balance between proinflammatory IFN-γ and TNF and anti-inflammatory interleukin-10 (IL-10) [3, 5].

One question is whether host genetics influence these responses. Racial differences, familial clustering, and murine studies support genetic control of leishmaniasis (reviewed in [6]). Human family-based genetic epidemiology of CL caused by *Leishmania peruviana*, a member of the *L. braziliensis* species complex, was consistent with a gene by environment multifactorial model, a 2-locus model of inheritance providing best fit [7]. This suggested that major genetic risk factors might be found for CL. Candidate gene studies [8–16] of *L. braziliensis* complex suggest that multiple genes associated with pro- and anti-inflammatory responses (*TNFA*, *SLC11A1*, *CXCR1*, *IL6*, *IL10*, *CCL2/MCP1*, *IFNG*) and/or wound healing (*FLI1*, *CTGF*, *TGFBR2*, *SMAD2*, *SMAD3*, *SMAD7*, *COL1A1*) influence CL or ML disease. Although frequently underpinned by functional data [12–14, 16] and/or supported by prior immunological studies [17, 18], these studies have generally lacked statistical power.

Here we perform the first well-powered genome-wide association study for *L. braziliensis* CL, combining analysis across 2 cohorts comprising 2066 cases and 2046 controls. Integrative post-GWAS analysis [19] is used to positionally map genomic loci associated with CL, with functional annotation and experimental studies used to identify plausible genes that act as genetic risk factors for CL.

## MATERIALS AND METHODS

### Ethical Considerations, Sampling, and Clinical Data Collection

The study, approved by the Hospital Universitário Professor Edgard Santos Ethical Committee (018/2008 and 22/2012) and the Brazilian National Ethical Committee (CONEP–305/2007; CONEP–1258513.1.000.5537), complied with principles of the Helsinki declaration. All participants or parents/guardians signed written consents. Post-quality control (QC) genotype data are lodged in the European Genome-Phenome Archive (accession number EGAS00001004596). CL cases were ascertained at the Public Health Post, Corte de Pedra, Bahia, Brazil, where *L. braziliensis* is the confirmed species [9–12]. CL is defined as presence of chronic ulcerative lesions without mucosal involvement (ML) or dissemination to ≥10 sites (DL). ML and DL cases were excluded due to insufficient power. All CL cases had confirmed parasite detection and/or minimally met 2 of 3 criteria: positive leishmania-specific DTH, positive leishmania serology, and leishmania histopathology. Endemic controls were attendants of cases with no current/previous history of CL, DL, or ML, including no scars. Samples were collected in 2 phases: 2008–2010 and 2016–2017. Blood bank controls were collected during 2015–2017 at the HEMOBA Foundation, Salvador. Demographic data (age, sex) were recorded. Blood (8 mL) was taken by venipuncture into dodecyl citrate acid–containing vacutainers (Becton Dickinson). Genomic DNA was prepared using proteinase K and salting-out and shipped to the United Kingdom for genotyping at Cambridge Genomic Services.

### Array Genotyping and Marker QC

DNAs were genotyped on Illumina Infinium HumanCoreExome Beadchips (Illumina, San Diego, California) with probes for 551 004 single-nucleotide variants (SNVs): 282 373 informative across ancestries; 268 631 exome-focused. Human genome build 37 (hg19) was used. Exclusions were individuals with missing data rate >5%, SNVs with genotype missingness >5%, minor allele frequency <0.01, or deviation from Hardy-Weinberg equilibrium (threshold *P* < 1.0 × 10^−8^). Post-QC datasets comprised 312 503 genotyped SNVs, 956 CL cases, 868 controls for phase 1; and 298 919 SNVs, 1110 CL cases, and 1178 controls for phase 2. Phases 1 and 2 had 52% and 81% power, respectively, the combined sample 99% power, to detect genome-wide significance (*P* < 5 × 10^−8^ [20]), assuming a disease allele frequency of 0.25, effect size (genotype relative risk) 1.5, and disease prevalence 2%.

### SNV Imputation and GWAS

Imputation was performed using the multiethnic 1000 Genomes Project phase 3 reference panel (1000G): 84.8 million variants, 2504 samples, and 26 populations. The 293 563 post-QC genotyped SNVs common across phases 1 and 2 were imputed using the Michigan Imputation Server version 1.0.4 [21]. Imputed SNVs with information metric <0.8 or genotype probability <0.9 were excluded. Remaining variants were converted to genotype calls and filtered for <5% missingness and minor allele frequency >0.005. Imputation accuracy was assessed as the squared Pearson correlation between imputed SNV dosage and known allele dosage (*r*^2^ > 0.5).

Genome-wide association analysis was performed using a linear mixed model in FaST-LMM version 2.07 under an additive model [22]. Population structure and relatedness were controlled using the genetic similarity matrix, computed from 32 696 phase 1 and 45 569 phase 2 linkage disequilibrium (LD)–pruned array variants. Systematic confounding was assessed using quantile-quantile (Q-Q) plots and an inflation factor (denoted λ; median observed/median theoretical χ ^2^ distributions). Manhattan plots were generated in R using mhtplot in the genetic analysis package “gap.” Regional association plots were created using LocusZoom [23]. The 32 696 phase 1 and 45 569 phase 2 LD-pruned variants were matched to HapMap populations and Principal Component Analysis plots prepared in R.

### Post-GWAS Annotation in FUMA

Functional Mapping and Annotation (FUMA) [19] was used to characterize regions of association based on positional, expression quantitative trait loci (eQTL) and chromatin interaction mapping. Summary statistics from the combined GWAS were loaded into FUMA. SNP2GENE was used to identify independent significant SNVs based on 1000G multiethnic LD data. SNP2GENE mapping used the default GWAS *P* < 10^−5^ plus 1 manually entered seed hit at *P* = 1.32 × 10^−5^. Independent significant SNVs and SNVs in LD with them were annotated for consequences on gene function using ANNOVAR, potential regulatory functions (Regulome DB score), and 15-core chromatin state predicted by ChromHMM for 127 tissue/cell types. Effects of SNVs on gene expression were determined using eQTLs from multiple tissue/cell types of healthy donors from databases: eQTLgen (44 different tissue types); BIOSQTL (BIO_eQTL_gene level, whole peripheral blood, 2116 healthy donors); DICE (B and T cells, monocytes, natural killer cells); and GTEx version 8 (whole blood; cultured fibroblasts; skin exposed and not sun exposed).

### Expression Analysis in CL Lesions

RNA expression for mapped genes was examined using published microarray data [24] comparing CL lesion biopsies (n = 25) with normal skin (n = 10) from nonendemic unexposed donors (GEO database: GSE55664). Between-group comparisons were made on log-transformed data using the GEO2R tool with Benjamini and Hochberg false discovery rate–adjusted *P* values.

### Cytokine and Antigen-stimulated T-Cell Responses

Plasma IFN-γ was measured using BioPlex-220 (Bio-Rad Laboratories) with Cytokine Grp-I-panel 27-plex. Peripheral blood mononuclear cells from a subset (n = 40) of untreated phase 2 CL patients were separated from heparinized blood over Ficoll and used to examine T-cell responses by *IFNG-AS1* genotype. Cells (1 × 10^6^ cells/mL) were stimulated: 37°C/5% CO_2_, 10 μg/mL *L. braziliensis* (strain MHOM/BR/2001) log-phase promastigote soluble *Leishmania* antigen, 1 μg/mL purified NA/LE anti-human CD28 (clone CD28.2, BD Biosciences, San Jose, California) for 15 hours, then brefeldin A (BD Biosciences) for 4 hours. Washed cells (phosphate-buffered saline/0.2% bovine serum albumin) were incubated (4°C; 30 minutes) with BUV661 anti-human CD3 monoclonal antibody (UCHT1 clone, BD Biosciences). Cells were fixed, washed, permeabilized using BD Cytofix/Cytoperm, and incubated (4°C; 30 minutes) with BV605 anti-human IFN-γ (B27 clone) and BUV395 anti-human TNF-α (MAB11 clone, BD Biosciences) in permeabilization buffer. Live/dead cells were distinguished using Fixable Viability Stain 575V (BD Biosciences). Data were acquired by BD FACSymphony A5 flow cytometry and analyzed using FlowJo 10.6.1 software (BD Biosciences), and differences between genotypes determined using nonparametric Kruskal-Wallis analysis of variance (ANOVA) with multiple comparisons.

## RESULTS

### Characteristics of the Study Population

Demographic and clinical details comparing cases and controls are provided in Supplementary Table 1. The younger age of endemic controls was counterbalanced by older age of blood bank controls. Phase 1 and 2 CL cases were matched for lesion number/size and DTH, with no correlation between lesion and DTH sizes. Blood bank controls fell within genetic heterogeneity of endemic controls (Supplementary Figures 1 and 2), with all controls matched to cases. A few outliers occurred in phase 1 endemic controls, which also showed greater heterogeneity in phase 2. Comparison against HapMap populations showed predominant admixture between white and African ethnicities. Linear mixed models used in association analyses take account of genetic heterogeneity.

### Genome-wide Association Study

**Figure 1.**
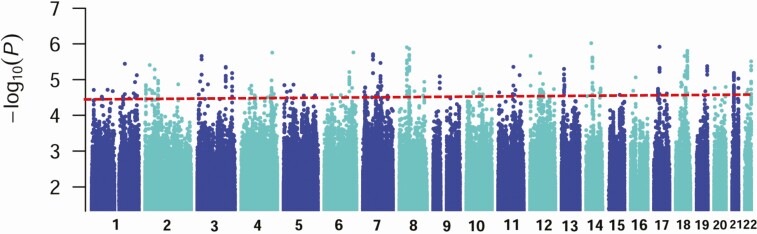
Manhattan plot of results from the combined analysis for the 4.46M high-quality 1000G imputed single-nucleotide variants (SNVs) common to phase 1 and phase 2 samples. Data are for analysis in FastLMM looking for association between SNVs and cutaneous leishmaniasis. The y-axis indicates −log_10_*P* values for association; the x-axis indicates the positions across each chromosome. The dotted line indicates the *P* = 5 × 10^−5^ cutoff used to look for suggestive associations.

Manhattan and Q-Q plots for genotyped data for phases 1/2 (Supplementary Figures 3 and 4) showed no systematic bias (λs 0.998/1). A Manhattan plot for the combined imputed genotype data (Figure 1) shows no hits at *P* < 5 × 10^−8^. Four approaches were used to identify susceptibility genes: (1) integrative post-GWAS annotation in FUMA [19]; (2) analysis of transcriptional data comparing lesions with normal skin [24]; (3) review of gene function for relevance to parasite biology/immunopathology; and (4) analysis of genotypic differences in T-cell responses.

### Integrative Post-GWAS Mapping and Annotation in FUMA

SNP2GENE identified 32 genomic loci associated with CL (Table 1; Supplementary Table 2). Positionally mapped SNVs localized to noncoding sequence, 58% intronic, 21% intergenic, 7% intronic in noncoding RNA genes, and 4% other. Most genomic loci (29/32) had a single lead SNV, with 5 additional independent significant SNVs at loci 9, 13, and 14. Top GWAS hits (=lead SNVs) at 23 loci were taken forward (Table 1): 18 at/near protein coding genes (21 genes: 15 intronic; 6 upstream/downstream), 5 intronic in noncoding RNA genes, and 1 intergenic <5 kb. Nine lead SNVs intergenic at >5 kb from the nearest gene were excluded from further consideration.

**Table 1.** Summary of SNP2GENE Results for Lead Genome-wide Association Study Single-nucleotide Variants and Associated Gene Information

Genomic Locus	Lead IndSigSNP^a^	rsID	Nearest Gene^b^	Type of Gene	Distance From Gene	Functional Location	No. of Pos^c^ Mapped SNVs	No. of eQTL SNVs	eQTL Database	eQTL Type^d^	Lesion vs Normal Skin^e^	Fold- change^f^
1	1:175280806	rs12753656	RP3-518E13.2: TNR	Antisense: protein coding	0	ncRNA intronic intronic	6	0	…	…	(TNR) NS	…
2	1:238427203	rs139144273	RP11-136B18.1	lincRNA	4590	Intergenic	1	0	…	…	ND	…
4	2:50706764	None	NRXN1	Protein coding	0	Intronic	40	0	…	…	NS	…
5	3:22117736	rs1383086	ZNF385D	Protein coding	0	Intronic	3	0	…	…	6.28 × 10–4	−1.8
6	3:149314267	rs536034590	WWTR1	Protein coding	0	Intronic	11	0	…	…	NS	
7	3:182857261	s74285558	**MCCC1**	Protein coding	231	Upstream	4	12	eQTLGen GTEx/v8 GTEx/v8 BIOSQTL	cis_eQTLs Skin SE Skin NSE Gene-level	7.98 × 10–10	−2.1
7	3:182857261	s74285558	**LAMP3**	Protein coding	0	Intronic	14	1	eQTLGen	cis_eQTLs	9.25 × 10–12	5.9
9	6:132815562	rs144488134	STX7	Protein coding	0	Intronic	6	0	…	…	0.008	1.3
12	7:93065079	rs143586968	CALCR	Protein coding	0	Intronic	8	0	…	…	3.62 × 10–4	1.7
13	8:40245200	rs125676	CTA-392C11.2	lincRNA	0	ncRNA intronic	47	0	…	…	ND	…
14	8:52628820	rs13261618	**PXDNL**	Protein coding	0	Intronic	62	0	…	…	NS	…
14	8:52628820	rs13261618	**PCMTD1**	Protein coding	0	Downstream	37	70	eQTLGenGTEx/v8	cis_eQTLs Fibroblasts	6.20 × 10–9	−1.9
16	11:80470102	None	RP11-686G23.2	lincRNA	0	ncRNA intronic	6	0	…	…	ND	…
18	12:3397404	rs77563142	TSPAN9	Protein coding	1673	Downstream	3	0	…	…	2.54 × 10–5	−1.8
19	12:52590004	rs10783496	KRT80	Protein coding	4219	Upstream	32	31	GTEx/v8; GTEx/v8; GTEx/v8	Fibroblasts Skin SE Skin NSE	3.07 × 10–8	−3.0
20	12:68407845	rs4913269	IFNG-AS1	Antisense	0	ncRNA intronic	10	10	eQTLGenGTEx/v8 BIOSQTL	cis_eQTLs Blood Gene-level	ND	…
22	14:47560881	rs6572403	MDGA2:MDGA2	Protein coding	0	Intronic	4	0	…	…	NS	…
23	14:53686046	rs1255253	AL163953.3	lincRNA	0	ncRNA intronic	52	0	…	…	ND	…
25	17:29136126	rs75270613	CRLF3	Protein coding	0	Intronic	8	0	…	…	9.19 × 10–9	2.0
26	18:46766154	rs4939853	DYM	Protein coding	0	Intronic	414	390	eQTLGenGTEx/v8 BIOSQTL	cis_eQTLs Blood Gene-level	NS	…
27	18:52955675	rs8090418	TCF4	Protein coding	0	Intronic	22	6	eQTLGen	cis_eQTLs	NS	…
28	18:61598763	rs8084306	SERPINB10	Protein coding	0	Intronic	31	28	eQTLGenGTEx/v8GTEx/v8	cis_eQTLs Skin SE Skin NSE	NS	…
29	19:55746886	rs12709949	PPP6R1	Protein coding	0	Intronic	19	0	…	…	6.09 × 10–7	1.7
31	21:48023640	rs201555201	S100B	Protein coding	0	Intronic	14	34	eQTLGenGTEx/v8 GTEx/v8 BIOSQTL	cis_eQTLs Skin SE Skin NSE Gene-level	NS	…
32	22:51038824	rs112974449	**CHKB**	Protein coding	0	Downstream	28	0	…	…	2.60 × 10–5	1.4
32	22:51038824	rs112974449	**MAPK8IP2**	Protein coding	0	Upstream	32	29	GTEx/v8 GTEX/v8	Skin SE Skin NSE	NS	…

Full details of genomic loci are provided in Supplementary Table 2.

Abbreviations: blood, whole blood; eQTL, expression quantitative trait locus; fibroblasts, cultured fibroblasts; ncRNA, noncoding RNA; ND, not done, NS, not significant; skin NSE, skin not sun exposed suprapubic; rsID, reference SNP cluster identity; SE, skin sun exposed lower leg; SNV, single-nucleotide variant; TNR, tenascin receptor.

^a^Lead IndSigSNP is the genome-wide association study top SNV.

^b^Bold indicates pairs of genes mapped with respect to the same Lead IndSigSNP.

^c^Pos indicates positionally mapped SNVs from SNP2GENE analysis.

^d^eQTL type/tissue-cell type.

^e^Analyzed from data in GEO database GSE55664 using the GEO2R tool with Benjamini and Hochberg false discovery rate–adjusted *P* values.

^f^Fold-change for GEO2R lesion vs normal skin analysis.

GWAS SNVs are generally enriched for eQTLs [25]. Focusing on data from tissues (whole blood, skin) and cell types (immune cells) relevant to CL (Table 1), SNP2GENE mapped eQTLs associated with expression of *MCCC1/LAMP3*, *PCMTD1*, *KRT80*, *IFNG-AS1*, *DYM*, *SERPINB10*, *S100B*, and *MAPK8IP2*.

### Transcriptional Analyses of Putative Susceptibility Genes

**Figure 2.**
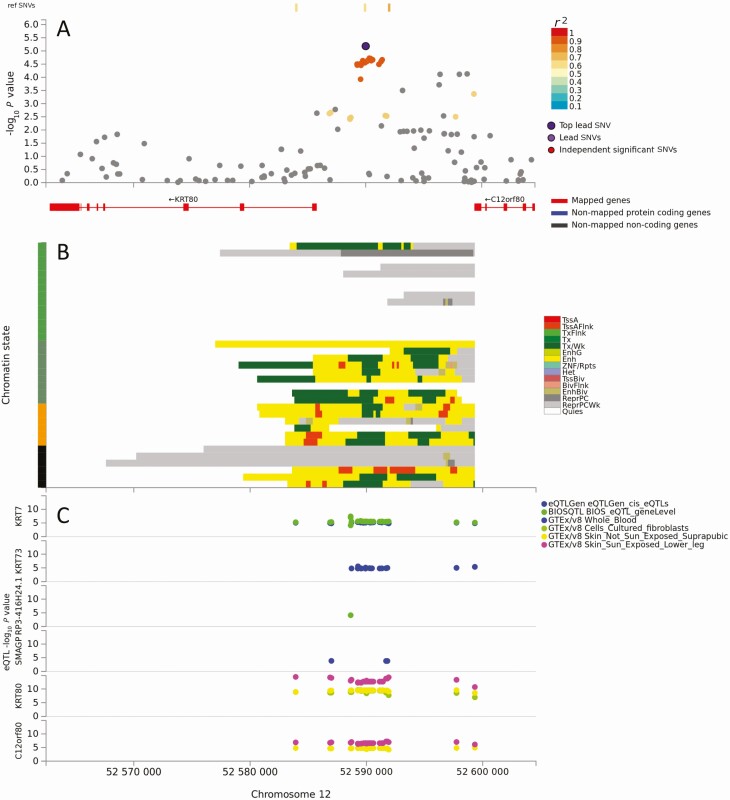
Results of positional, chromatin interaction, and expression quantitative trait locus (eQTL) activity mapping in functional mapping and annotation for *KRT80*. *A*, Map showing the top lead single-nucleotide variant (SNV), and SNVs in linkage disequilibrium with it according to the *r*^2^ color-coded key, across the 2 genes. There were no additional independent significant SNVs. *B*, Chromatin-15 states color coded for transcriptional/enhancer activity as shown in the key. The y-axis color coding relates to cell/tissue types in which chromatin interaction was mapped. *C*, eQTL activity for genes (y-axis) in different cells/tissues from public domain databases as shown in the key. Full explanation of keys provided as preamble to the supplementary figures. Color figure available online.

Additional evidence (Table 1) to support genes as candidates was sought by comparing expression in CL lesions vs normal skin [24]. Six genes were expressed at higher level in lesions (*LAMP3*, *STX7*, *CALCR*, *CRLF3*, *PPP6R1*, *CHKB*), 5 genes at lower levels (*ZNF385D*, *MCCC1*, *PCMTD1*, *TSPAN9*, *KRT80*). For 5 differentially expressed genes, *ZNF385D*, *STX7*, *CALCR*, *TSPAN9*, *PPP6R1*, SNP2GENE eQTL mapping provided no evidence for SNVs associated with expression in selected tissues (whole blood, skin) or cell types (fibroblasts, immune cells). A role for GWAS SNVs regulating expression of *KRT80* was supported by eQTL and chromatin interaction mapping (Figure 2). The lead SNV and others in strong LD lie upstream of *KRT80* in a region of strong transcriptional/enhancer activity and act as eQTLs in cultured fibroblasts, sun and non-sun-exposed skin. Other genes supported by both eQTL and lesion expression were in tandem with genes positionally mapped to the same lead SNV (Table 1), namely *LAMP3/MCCC1*, *PXDNL/PCMTD1*, and *CHKB/MAPK8IP2*. For *PXDNL/PCMTD1*, the lead SNV was intronic in *PXDNL* but neither it nor numerous mapped SNVs in LD with it were eQTL for *PXDNL* itself. Rather, they acted as eQTLs for *PCMTD1* expression in fibroblasts (Supplementary Figure 5). For *CHKB*/*MAPK8IP2*, the lead SNV lies upstream of both genes transcribed in opposing directions. Mapped SNV act as eQTLs for *MAPK8IP2* in sun and not-sun-exposed skin but not for *CHKB* (Supplementary Figure 6). For *LAMP3*/*MCCC1*, the lead SNV and SNVs in LD with it map predominantly within *LAMP3* (Supplementary Figure 7). While they act as cis-eQTLs for *MCCC1* expression, data from CL lesions (Table 1) suggest stronger upregulation of *LAMP3* compared to downregulation of *MCCC1*.

**Figure 3.**
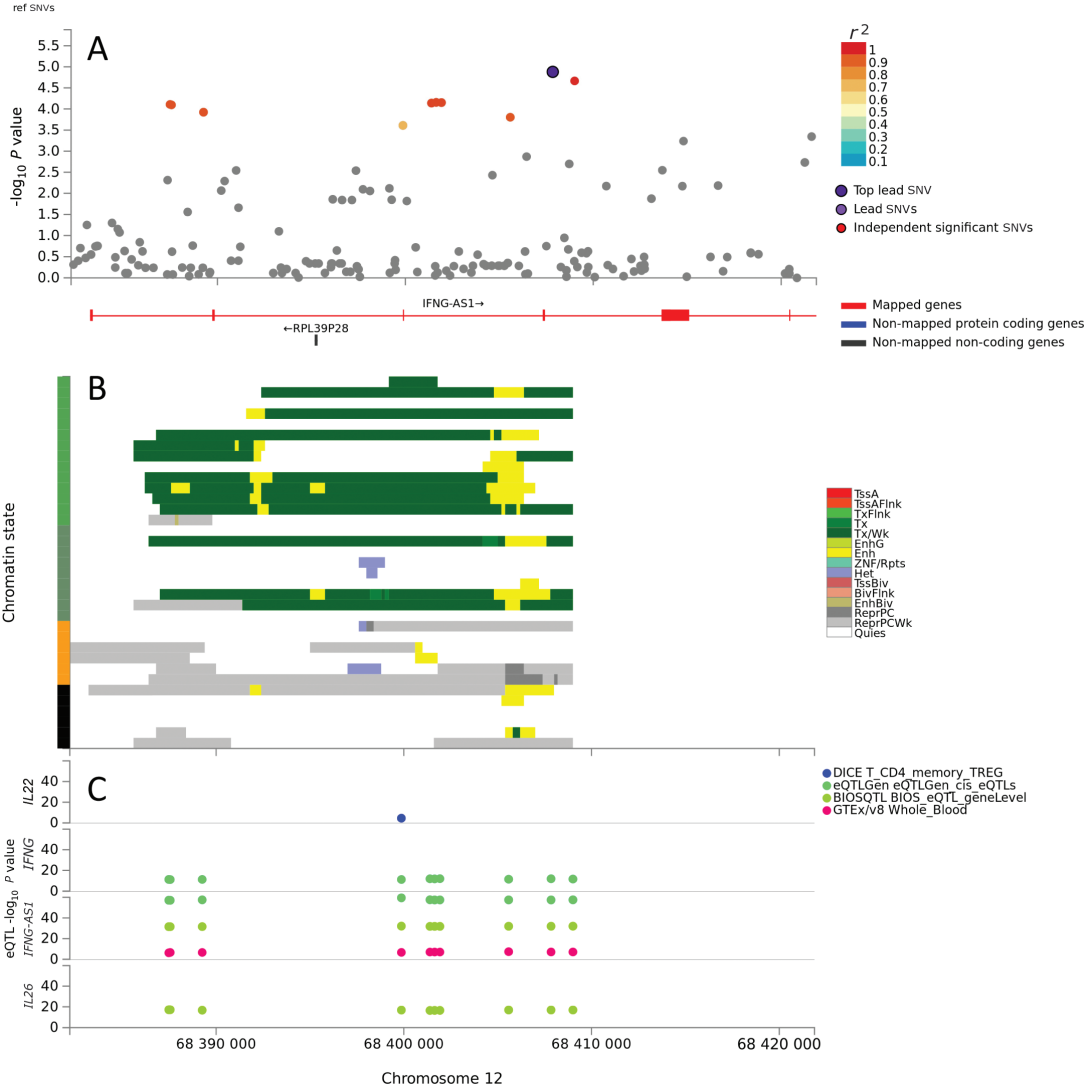
Results of positional, chromatin interaction, and expression quantitative trait locus (eQTL) activity mapping in functional mapping and annotation for *IFNG-AS1*. *A*, Map showing the top lead single-nucleotide variant (SNV), and SNVs in linkage disequilibrium with it according to the *r*^2^ color-coded key, across the 2 genes. There were no additional independent significant SNVs. *B*, Chromatin-15 states color coded for transcriptional/enhancer activity as shown in the key. The y-axis color coding relates to cell/tissue types in which chromatin interaction was mapped. *C*, eQTL activity for genes (y-axis) in different cells/tissues from public domain databases as shown in the key. Full explanation of keys provided as preamble to supplementary figures. Color figure available online.

Ten genes showed no differential expression in lesions (Table 1). This included *SERPINB10* across which multiple cis-eQTLs mapped to chromatin states of transcriptional/enhancer activity in neutrophils, monocytes, B cells, and hematopoietic stem cells (Supplementary Figure 8). Four noncoding RNA genes (Table 1) not present on the chips used for CL lesion data [24] included *IFNG-AS1* which had 10 eQTLs across a chromatin state region of transcriptional/enhancer activity in immune cells (whole blood: T cells, B cells, hematopoietic stem cells) that were associated with expression of *IFNG-AS1*, *IFNG*, and *IL26* (Figure 3).

### Relevance to the Biology and Immunopathology of CL Disease

**Figure 4.**
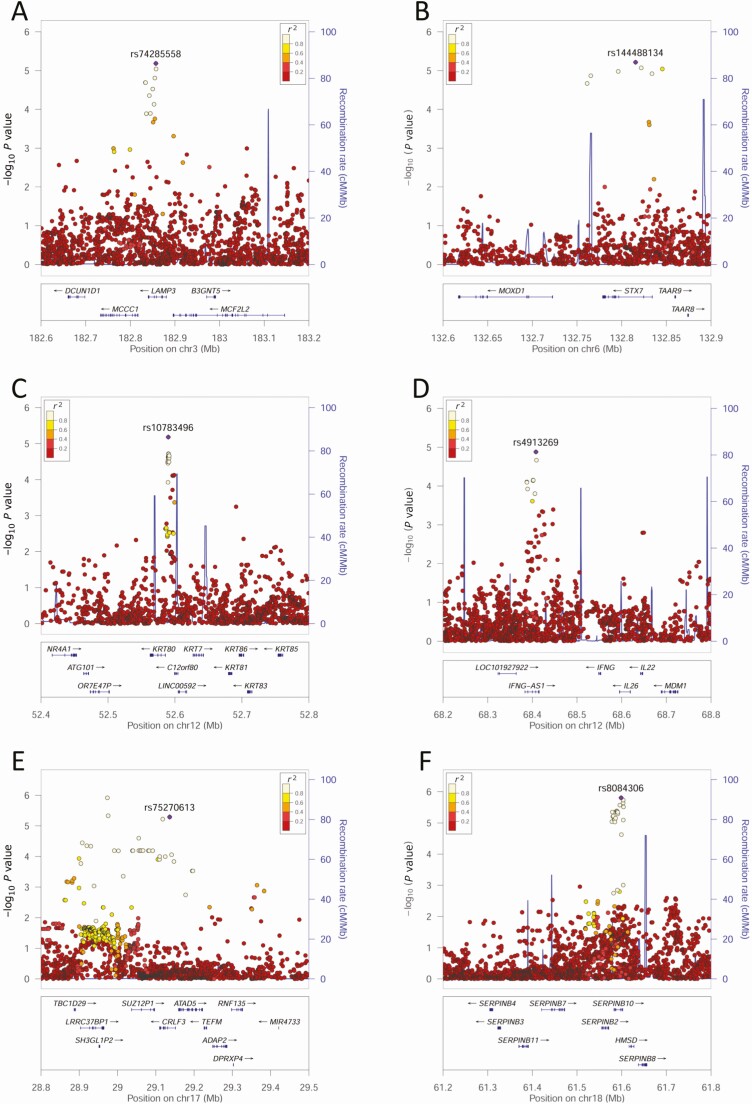
LocusZoom plots for genome-wide association study (GWAS) associations identified as plausible genetic risk factors for cutaneous leishmaniasis following post-GWAS annotation: *LAMP3* (*A*), *STX7* (*B*), *KRT80* (*C*), *CRLF3* (*D*), *SERPINB10* (*E*), and *IFNG-AS1* (*F*). The −log_10_*P* values (left y-axis) are shown in the top section of each plot. Dots representing individual single-nucleotide variants (SNVs) are color coded/grey-scale shaded (see key) based on their population-specific linkage disequilibrium *r*^2^ with the top SNV (annotated by rsID [reference SNP cluster identity]) in the region. The right y-axis is for recombination rate (blue line), based on HapMap data. The bottom section of each plot shows the positions of genes across the region.

In summary, of 32 positional mapped genomic loci, 23 occurred at protein coding or noncoding RNA genes of which 15 had eQTLs for expression in relevant cells/tissues and/or showed differential expression in CL lesions. To determine which genes in these 15 loci might act as CL susceptibility genes, we reviewed gene function in relation to parasite biology and CL immunopathology (Supplementary Table 3). A plausible functional role for 12 genes was not found, including *PCMTD1* for which eQTL and lesion expression data were strong. A role for these genes cannot be discounted, but 6 genes had plausible links to CL pathogenesis (Table 2 and Figure 4): *LAMP3* and *STX7* play a role in lysosome function; *KRT80* and *CRLF3* relate to skin perturbations; *SERPINB10* and *IFNG-AS1* play central roles in immune responses.

**Table 2.** Top Genome-wide Association Study Hits in Genes of Plausible Functional Interest as Genetic Risk Factors for Cutaneous Leishmaniasis Caused by *Leishmania braziliensis*

Chr	Position, bp	rsID	*P* Value	Odds Ratio (95% CI)	Beta (SE)	Allele^a^	Variant Origin	Location	Gene	Function
3	182857261	rs74285558	6.54 × 10–6	0.87 (.82–.92)	−.034 (0.008)	T (C/T)	Global	intron	*LAMP3*	Lysosomal associated membrane protein 3
6	132815562	rs144488134	6.10 × 10–6	0.82 (.75–.89)	−.034 (0.007)	A (C/A)	African	intron	*STX7*	Syntaxin 7
12	52590004	rs10783496	6.58 × 10–6	1.06 (1.03–1.09)	.035 (0.008)	A (G/A)	Global	intron	*KRT80*	Keratin 80
12	68407845	rs4913269	1.32 × 10–5	1.06 (1.03–1.08)	.033 (0.008)	G (C/G)	Global	intron	*IFNG-AS1*	IFNG antisense RNA 1
17	29136126	rs75270613	5.12 × 10–6	0.83 (.77–.90)	−.034 (0.008)	T (C/T)	African	intron	*CRLF3*	Cytokine receptor like factor 3
18	61598763	rs8084306	1.56 × 10–6	1.07 (1.04–1.10)	.038 (0.008)	C (T/C)	Global	intron	*SERPINB10*	Serpin family B member 10

Details of all post–genome-wide association study candidate genes are provided in Supplementary Table 3.

Abbreviations: Chr, chromosome; CI, confidence interval; rsID, reference SNP cluster identity; SE, standard error.

^a^Associated allele (ancestral/minor) for risk or protection as indicated by the odds ratio.

### Relating *IFNG-AS1* Genotypes to Antigen-specific T-Cell Responses

**Figure 5.**
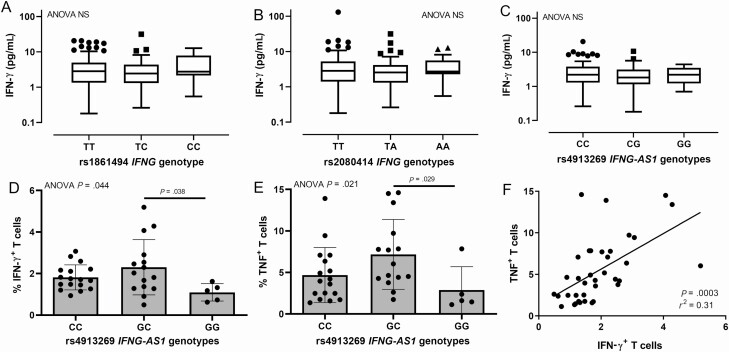
Plots examining *IFNG* and *IFNG-AS1* genotypes by interferon gamma (IFN-γ) and tumor necrosis factor (TNF) responses. *A–C*, Results for plasma levels of IFN-γ. *A* and *B*, There is no association between plasma IFN-γ and genotypes for 2 single-nucleotide variants (SNVs) at *IFNG*, rs1861494 that was associated with cutaneous leishmaniasis (CL) disease for *Leishmania guyanensis* in a previous study [16] and rs2080414 that was in the strongest linkage disequilibrium with rs2069705 that was associated with CL disease and plasma IFN-γ in that study (rs2069705 was not genotyped or imputed in the present study). *C*, There is no association between plasma IFN-γ and rs4913269 at *IFNG-AS1*. *D* and *E*, Differences in percentages of antigen-stimulated IFN-γ and TNF-producing CD3^+^ T cells by *IFNG-AS1* genotype for the top SNV rs4913269 at chromosome 12 bp position 68407845 associated with CL disease in our study. *F*, Correlation between percentage IFN-γ ^+^ and percentage TNF^+^ CD3^+^ T cells for individuals genotyped. Abbreviations: ANOVA, analysis of variance; IFN-γ, interferon gamma; NS, not significant; TNF, tumor necrosis factor.

The GWAS (Supplementary Data) showed modest support (*P* < .01) for some previous candidate genes, but not for *IFNG* variants associated with *Leishmania guyanensis* CL in Brazil [16], including no association with plasma IFN-γ (Figure 5A and 5B). *IFNG-AS1* expression influences IFN-γ production [26]. While eQTL mapping supported SNVs at *IFNG-AS1* acting as cis-eQTLs for *IFNG* and *IL26*, they were stronger eQTLs for *IFNG-AS1* (Figure 3). Plasma IFN-γ did not differ by *IFNG-AS1* genotype (Figure 5C), but a significant difference in the percentage of antigen-specific IFN-γ–producing T cells across genotypes was observed at rs4913269 (ANOVA *P*_adjusted_ = .044) (Figure 5D). Individuals homozygous for the disease-associated G allele showed a significantly lower percentage of IFN-γ–producing T cells compared with heterozygotes. Similar genotype associations occurred for TNF-producing T cells (Figure 5E; ANOVA *P*_adjusted_ = .021), which were strongly correlated with IFN-γ–producing T cells (Figure 5F; *r*^2^ = 0.31; *P* = .0003). Parallel observations were made for 6 other SNVs in strong LD (Figure 4D) with rs4913269.

## DISCUSSION

Our GWAS provides the first hypothesis-free insights into genetic risk factors for *L. braziliensis* CL. Despite prior evidence for genetic regulation of leishmaniasis [7], and the robust well-powered study undertaken, no signals of association achieved genome-wide significance (*P* < 5 × 10^−8^) and only modest support was found for previous candidate gene studies (Supplementary Data). We therefore employed integrative approaches [19] to prioritize 6 genes as plausible genetic risk loci for CL.

Two genes relate to intracellular localization of *Leishmania* parasite in phagolysosomes [27]. *LAMP3* encodes lysosomal-associated membrane protein 3, also known as dendritic cell LAMP or DCLAMP [28]. Expression of DCLAMP increases in activated dendritic cells, localizing to the MHC class II compartment immediately before translocation of class II to the cell surface [28]. LAMP3 was expressed at 5.9-fold higher levels in lesions compared to normal skin, supporting its role in CL. Since dendritic cells are the most potent antigen-presenting cells that induce primary T-cell responses, it is likely that variation at *LAMP3* will relate to presentation of *Leishmania* antigens to T cells. *STX7* encodes syntaxin 7, which influences vesicle trafficking to lysosomes, including phagosome-lysosome fusion [29]. Variants at *STX7* could influence delivery of *Leishmania* phagosomes to lysosomes of macrophages.

Two other susceptibility genes, *KRT80* and *CRLF3*, likely relate to skin perturbations and/or the wound healing response. Keratin 80 is a type II epithelial keratin with biased expression in skin keratinocytes [30]. Pathogens invading skin cause keratinocytes to produce chemokines which attract monocytes, natural killer cells, T cells, and dendritic cells [31]. *KRT80* and multiple other keratins (*KRT77/81/4/39/32/33B*) were expressed at lower levels in lesions compared to normal skin. Whether this reflects a paucity of keratinocytes in lesions, or specific downregulation of gene expression in keratinocytes within lesions, requires further investigation. Keratinocytes play a role in wound healing, are potent producers of IL-10 and transforming growth factor–β [32], and can change to a sclerotic phenotype by gene silencing of *Fli1* [33]. Although association of human CL and *FLI1* [9, 10] was not replicated here, *Fli1* is a confirmed murine CL susceptibility gene [34]. Our novel associations continue to focus on molecules/cells involved in wound healing. In contrast, the cytokine receptor-like factor 3 *CRLF3* is expressed in normal skin, and shows pathologically enhanced expression in premalignant actinic keratosis and malignant squamous cell carcinoma [35]. *CRLF3* appears to be similarly dysregulated in CL lesions.

*SERPINB10* and *IFNG-AS1* are highlighted as central regulators of immune responses. Serpin family B member 10 is a peptidase inhibitor expressed in bone marrow [36] in the monocytic lineage and can inhibit TNF-induced apoptosis [37]. Epithelial SERPINB10 contributes to allergic eosinophilic inflammation [38]. However, despite eQTL data showing SERPINB10 expressed in sun-exposed skin, we found no evidence for its expression in lesions, consistent with more central roles in immune regulation. IFNG antisense RNA 1 fine-tunes the magnitude of IFN-γ responses [26]. It is expressed in mouse and human T-helper1 cells and positively regulates *Ifng* expression [39, 40]. Transient overexpression of *Ifng-as1* is associated with increased IFN-γ and reduced susceptibility to *Salmonella enterica* [39]. Conversely, deletion of *Ifng-as1* in mice compromises host defence against *Toxoplasma gondii* by reducing *Ifng* expression. Discordant expression of *IFNG* and *IFNG-AS1* is seen in long-lasting memory T cells, where high *IFNG-AS1* associated with low *IFNG* suggests feedback inhibition [26]. We observed that the *IFNG-AS1* genotype was associated with downstream effects on percentage of IFN-γ–producing CD3^+^ T cells and highly correlated TNF-producing CD3^+^ T cells following antigen stimulation. Individuals homozygous for the disease-associated allele at 7 *IFNG-AS1* associated SNVs had significantly lower percentages of IFN-γ/TNF T cells, suggesting that lower IFN-γ and TNF regulated by *IFNG-AS1* causes increased disease risk. These 2 proinflammatory cytokines are important activators of macrophages for anti-leishmanial activity.

Our GWAS identified novel genetic risk factors for CL that provide interesting leads to further understanding CL pathogenesis, including through regulation of IFN-γ responses.

## Supplementary Data

Supplementary materials are available at *Clinical Infectious Diseases* online. Consisting of data provided by the authors to benefit the reader, the posted materials are not copyedited and are the sole responsibility of the authors, so questions or comments should be addressed to the corresponding author.

ciaa1230_suppl_Supplementary_Figures

ciaa1230_suppl_Supplementary_Tables

ciaa1230_suppl_Supplementary_Information

## Notes

***Acknowledgments.*** The authors thank the staff of the Health Post at Corte de Pedra for their assistance in the collection of samples and clinical and field data, as well as staff at the HEMOBA Foundation Blood Bank in Salvador.

***Disclaimer.*** The study sponsor had no role in study design, data collection, data analysis, data interpretation, or writing of the report. The corresponding author had full access to all study data and had final responsibility for the decision to submit for publication.

***Financial support.*** This work was supported by the British Medical Research Council (MRC) grant number MR/N017390/1; a Brazilian Fundação de Amparo à Pesquisa do Estado de Minas Gerais (FAPEMIG) grant in cooperation with MRC/Conselho Nacional das Fundações Estaduais de Amparo à Pesquisa (CONFAP) (CBB-APQ-00883-16); National Institute of Science and Technology in Tropical Diseases, Brazil (grant number 573839/2008–5); CNPq (K. J. G. and W. O. D. are CNPq fellows); Fundação de Amparo à Pesquisa do Estado de São Paulo (FAPESP) (Fellowships for N. S. A. and A. B. F.); the National Institute of Science and Technology in Tropical Diseases, Brazil (number 573839/2008–5); and the National Institutes of Health (grant number AI 30639). H. J. C. and S. C. were supported by the Wellcome Trust (grant number 102858/Z/13/Z).

***Potential conflicts of interest.*** The authors: No reported conflicts of interest. All authors have submitted the ICMJE Form for Disclosure of Potential Conflicts of Interest.
