# Supplementary material for: A Genome-wide Association Study Identifies SERPINB10, CRLF3, STX7, LAMP3, IFNG-AS1, and KRT80 As Risk Loci Contributing to Cutaneous Leishmaniasis in Brazil
Source: Clin Infect Dis. 2020 Aug 23;72(10):e515–25. doi: 10.1093/cid/ciaa1230 (PMC8130031; doi:10.1093/cid/ciaa1230)
Supplement: ciaa1230_suppl_Supplementary_Figures [file ciaa1230_suppl_supplementary_figures.pdf]

A Genome-Wide Association Study Highlights a Regulatory Role for *IFNG-AS1* Contributing to Cutaneous Leishmaniasis In Brazil

Léa C. Castellucci,[1,2,*] Lucas Almeida,[1,2,*] Svetlana Cherlin,[3,*] Michaela Fakiola,[4,*,†] Richard W. Francis,[5] Edgar M. Carvalho,[1] Anadílton Santos da Hora,[2] Tainã Souza do Lago,[2] Amanda B. Figueiredo,[6] Clara M. Cavalcanti,[6] Natalia S. Alves,[6] Katia LP Morais.[6] Andréa Teixeira-Carvalho,[7] Walderez O. Dutra,[1,8] Kenneth J. Gollob,[1,6,9] Heather J. Cordell,[3] and Jenefer M. Blackwell[5,10] [*]Contributed equally

[1]National Institute of Science and Technology in Tropical Diseases, Brazil; [2]Federal University of Bahia, Salvador, Brazil; [3]Population Health Sciences Institute, Newcastle University, UK; [4]INGM-National Institute of Molecular Genetics "Romeo ed Enrica Invernizzi" Milan, Milan, Italy; [5]Telethon Kids Institute, The University of Western Australia, Western Australia; [6]International Center for Research, AC Camargo Cancer Center, São Paulo, Brazil; [7]Instituto Rene Rachou of Fundação Oswaldo Cruz (FIOCRUZ-Minas), Belo Horizonte, Brazil; [8]Instituto de Ciências Biológicas, Universidade Federal de Minas Gerais, Belo Horizonte, Brazil; [9]Núcleo de Ensino e Pesquisa, Instituto Mario Penna, Belo Horizonte, Brazil; [10]Department of Pathology, University of Cambridge, UK; [†]Present address: IFOM, the FIRC Institute of Molecular Oncology, Milan, Italy

Corresponding authors: Jenefer M. Blackwell (jenefer.blackwell@telethonkids.org.au) and Kenneth Gollob (kenneth.gollob@accamargo.org.br)

Supplementary Figures

Preamble: Information from the summary of genome-wide SNP2GENE results obtained using FUMA are provided here along with keys to aid in interpretation of main Figures 2 and 3, and Supplementary Figures 5 to 8.

Summary of SNPs and mapped genes

#Genomic risk loci	32
#lead SNPs	32
#Ind. Sig. SNPs	37
#candidate SNPs	1102
#candidate GWAS tagged SNPs	950
#mapped genes	108

Functional consequences of SNPs on genes ?

ℹ Statistics are available in "annov.stats.txt". The file is downloadable from the "Download" tab.
Download the plot as PNG JPG SVG PDF

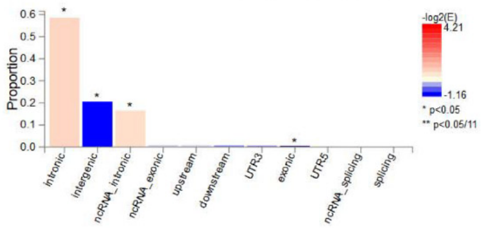

Summary per genomic risk locus ?

Download the plot as PNG JPG SVG PDF

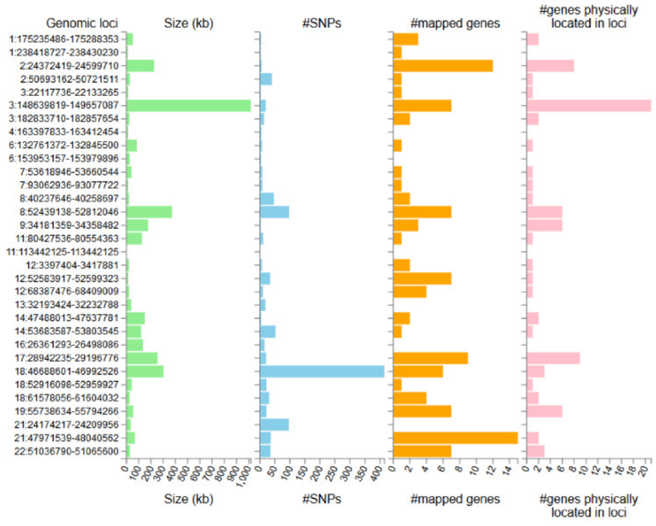

A. Positional mapping:

Shown in Parts A of each of main Figures 2 and 3, and Supplementary Figures 5 to 8

Red: Mapped genes. Genes mapped by positional mapping are always colored red.
Blue: Non-mapped protein-coding genes.
Dark grey: Non-mapped non-coding genes.
GWAS P-value: SNVs (also known as SNPs) which are not in LD of any of significant independent lead SNPs in the selected region are colored grey.

B. ROADMAP Chromatin States in 15-state models:

https://egg2.wustl.edu/roadmap/web_portal/chr_state_learning.html

Shown in Parts B of each of main Figures 2 and 3, and Supplementary Figures 5 to 8

127 epigenomes, 5 chromatin marks

- H3K4me3
- H3K4me1
- H3K36me3
- H3K27me3
- H3K9me3

15-core chromatin state data for Blood and Skin – Y-axis* colour codes for epigenomes used in chromatin state mapping.

Epigenome ID				
EID	Color	Group	Anatomy	Standerdized epigenome name
E062	#55A354	Blood & T-cell	BLOOD	Primary mononuclear cells from peripheral blood
E034	#55A354	Blood & T-cell	BLOOD	Primary T cells from peripheral blood
E045	#55A354	Blood & T-cell	BLOOD	Primary T cells effector/memory enriched from peripheral blood
E033	#55A354	Blood & T-cell	BLOOD	Primary T cells from cord blood
E044	#55A354	Blood & T-cell	BLOOD	Primary T regulatory cells from peripheral blood
E043	#55A354	Blood & T-cell	BLOOD	Primary T helper cells from peripheral blood
E039	#55A354	Blood & T-cell	BLOOD	Primary T helper naive cells from peripheral blood
E041	#55A354	Blood & T-cell	BLOOD	Primary T helper cells PMA-I stimulated
E042	#55A354	Blood & T-cell	BLOOD	Primary T helper 17 cells PMA-I stimulated
E040	#55A354	Blood & T-cell	BLOOD	Primary T helper memory cells from peripheral blood 1
E037	#55A354	Blood & T-cell	BLOOD	Primary T helper memory cells from peripheral blood 2
E048	#55A354	Blood & T-cell	BLOOD	Primary T CD8+ memory cells from peripheral blood
E038	#55A354	Blood & T-cell	BLOOD	Primary T helper naive cells from peripheral blood
E047	#55A354	Blood & T-cell	BLOOD	Primary T CD8+ naive cells from peripheral blood
E029	#678C69	HSC & B-cell	BLOOD	Primary monocytes from peripheral blood
E031	#678C69	HSC & B-cell	BLOOD	Primary B cells from cord blood
E035	#678C69	HSC & B-cell	BLOOD	Primary hematopoietic stem cells
E051	#678C69	HSC & B-cell	BLOOD	Primary hematopoietic stem cells G-CSF-mobilized Male
E050	#678C69	HSC & B-cell	BLOOD	Primary hematopoietic stem cells G-CSF-mobilized Female
E036	#678C69	HSC & B-cell	BLOOD	Primary hematopoietic stem cells short term culture
E032	#678C69	HSC & B-cell	BLOOD	Primary B cells from peripheral blood
E046	#678C69	HSC & B-cell	BLOOD	Primary Natural Killer cells from peripheral blood
E030	#678C69	HSC & B-cell	BLOOD	Primary neutrophils from peripheral blood
E055	#FF9D0C	Epithelial	SKIN	Foreskin Fibroblast Primary Cells skin01
E056	#FF9D0C	Epithelial	SKIN	Foreskin Fibroblast Primary Cells skin02
E059	#FF9D0C	Epithelial	SKIN	Foreskin Melanocyte Primary Cells skin01
E061	#FF9D0C	Epithelial	SKIN	Foreskin Melanocyte Primary Cells skin03
E057	#FF9D0C	Epithelial	SKIN	Foreskin Keratinocyte Primary Cells skin02
E058	#FF9D0C	Epithelial	SKIN	Foreskin Keratinocyte Primary Cells skin03
E115	#000000	ENCODE2012	BLOOD	Dnd41 TCell Leukemia Cell Line
E116	#000000	ENCODE2012	BLOOD	GM12878 Lymphoblastoid Cells
E123	#000000	ENCODE2012	BLOOD	K562 Leukemia Cells
E124	#000000	ENCODE2012	BLOOD	Monocytes-CD14+ RO01746 Primary Cells
E126	#000000	ENCODE2012	SKIN	NHDF-Ad Adult Dermal Fibroblast Primary Cells
E127	#000000	ENCODE2012	SKIN	NHEK-Epidermal Keratinocyte Primary Cells

*The order of the cell types is same as this legend table.

Key to colour coded regions on X-axis:

- Active TSS
- Flanking Active TSS
- Transcr. at gene 5' and 3'
- Strong transcription
- Weak transcription
- Genic enhancers
- Enhancers
- ZNF genes & repeats
- Heterochromatin
- Bivalent/Poised TSS
- Flanking Bivalent TSS/Enh
- Bivalent Enhancer
- Repressed PolyComb
- Weak Repressed PolyComb
- Quiescent/Low

C. eQTLs

Shown in Parts B of each of main Figures 2 and 3, and Supplementary Figures 5 to 8

The color of eQTLs for different databases shown in the graph key is assigned arbitrarily. All eQTLs with user defined P-value threshold and tissue types are displayed.

Supplementary Figure 1

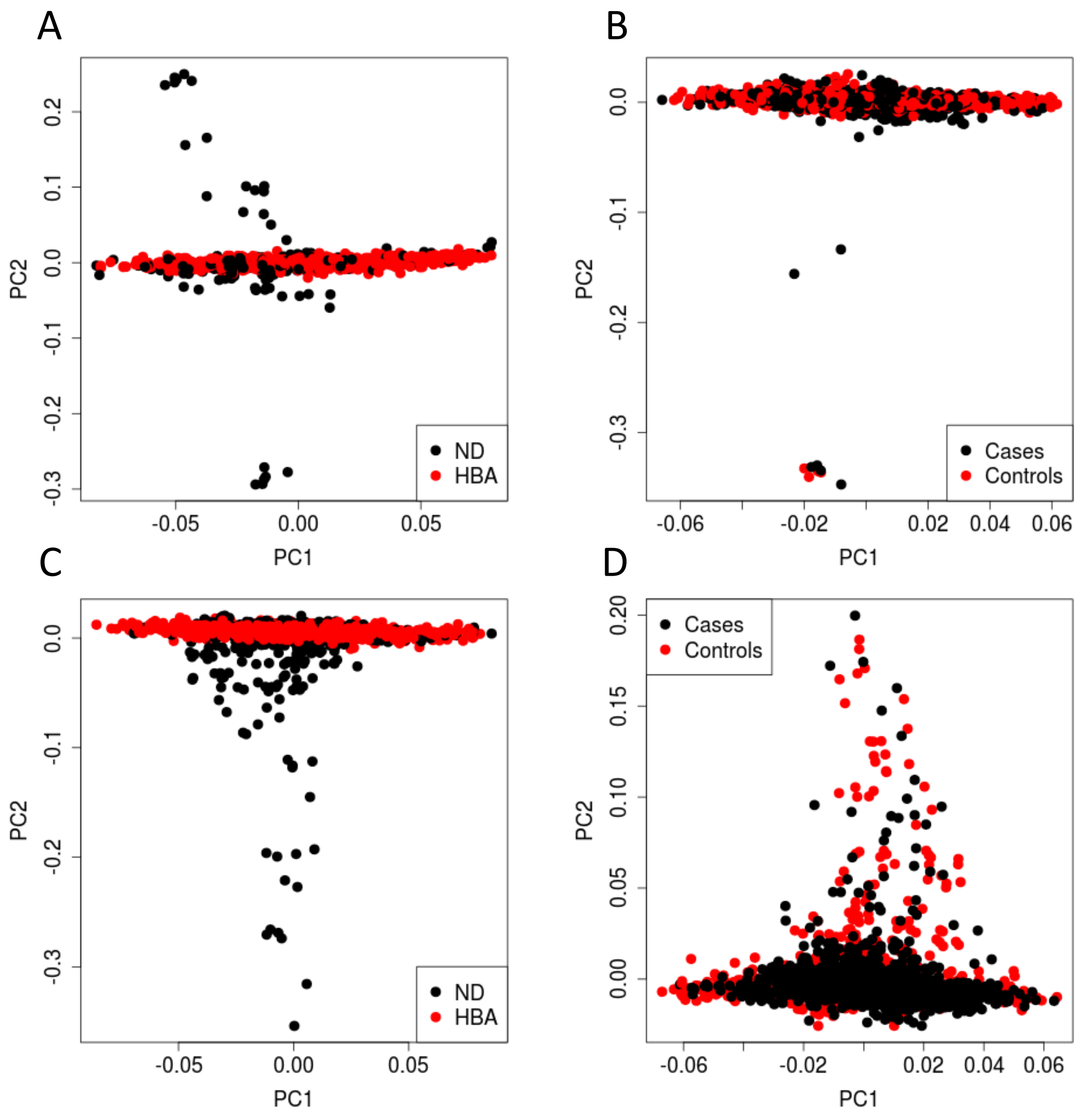

Supplementary Figure 1. PCA plots comparing genetic heterogeneity across cases and controls used in the study. (A) and (C) compare endemic control (ND) against blood bank controls (HBA) for Phase 1 and Phase 2 samples, respectively. (B) and (D) compare all cases against all controls for Phase 1 and Phase 2 samples, respectively.

Supplementary Figure 2

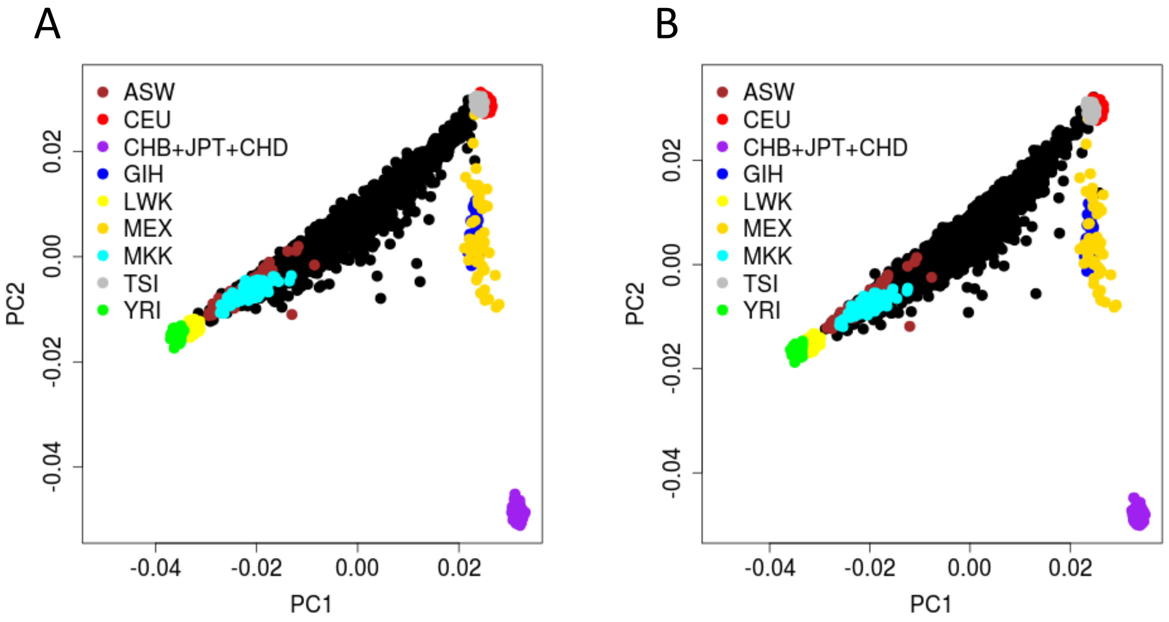

Supplementary Figure 2. PCA plots comparing genetic heterogeneity in the study sample against HapMap control populations. (A) shows the plot for all Phase 1 samples; (B) shows the plot for all Phase 2 samples. Abbreviations for HapMap populations: ASW - Americans of African Ancestry in SW US; CEU - Utah Residents (CEPH) with Northern and Western European Ancestry; CHB - Han Chinese in Beijing, China; JPT - Japanese in Tokyo, Japan; CHD - Chinese in Metropolitan Denver, Colorado; GIH - Gujarati Indian from Houston, Texas; LWK - Luhya in Webuye, Kenya; MEX - Mexican ancestry in Los Angeles, California; MKK - Maasai in Kinyawa, Kenya; TSI - Toscani in Italia; YRI - Yoruba in Ibadan, Nigeria.

Supplementary Figure S3

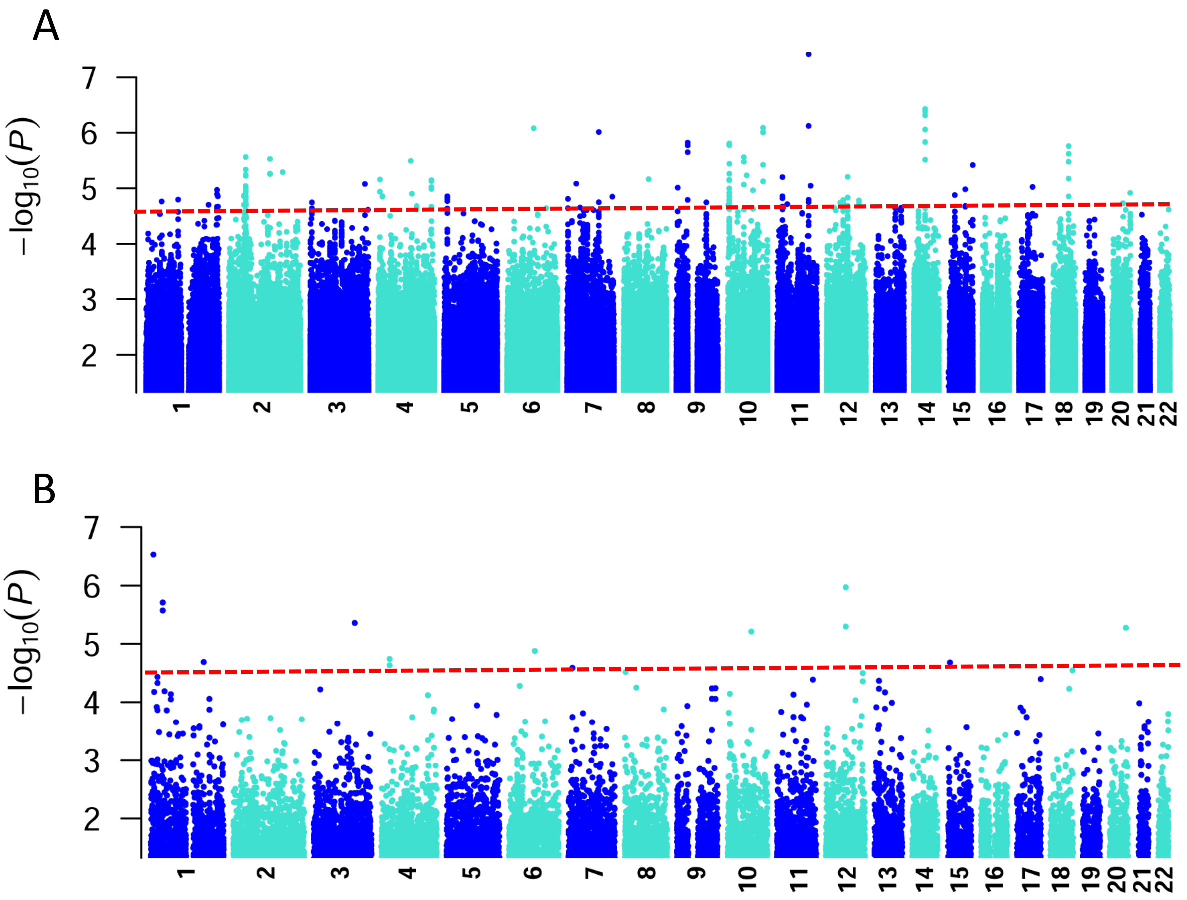

Figure S3. Manhattan plots of results of association analyses for genotyped SNVs. (A) for Phase 1. (B) for Phase 2. Data are for analysis in FastLMM looking for association between SNVs and CL. The Y-axis indicates –log10 *P* values for association, the X axis indicates the positions across each chromosome. The red dotted lines indicate the P=5x10^{-5} cut-off used to look for suggestive associations in the combined analysis (main Figure 1).

Supplementary Figure S4

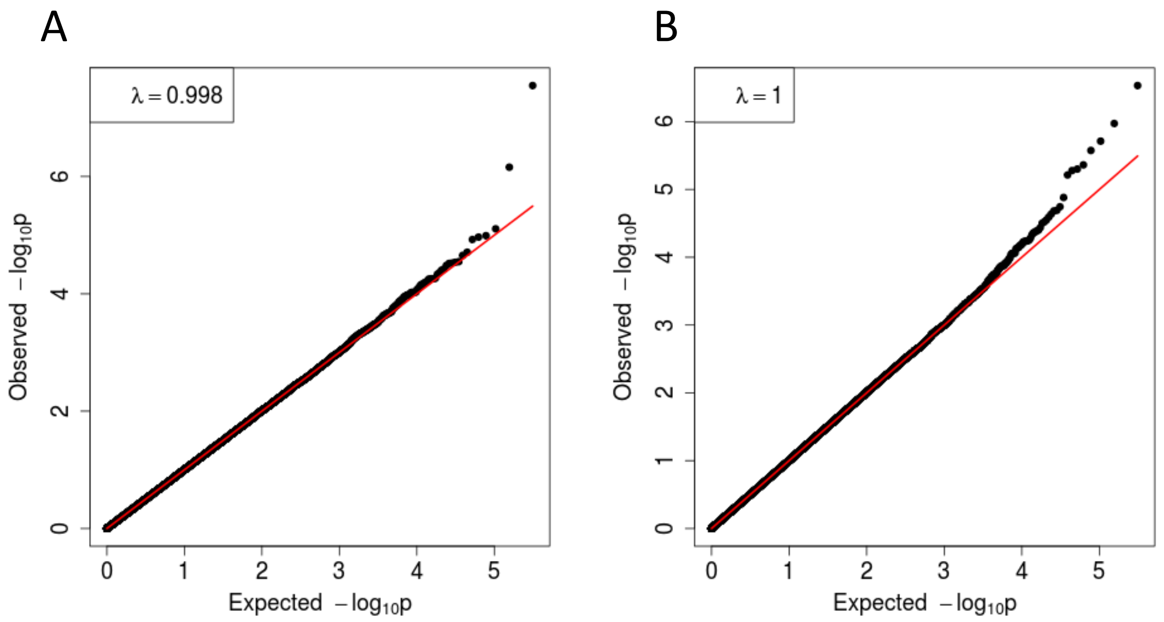

Figure S4. Quantile-quantile plot of GWAS p-values for genotyped data for (A) Phase 1; and (B) Phase 2 of the study.

Supplementary Figure S5

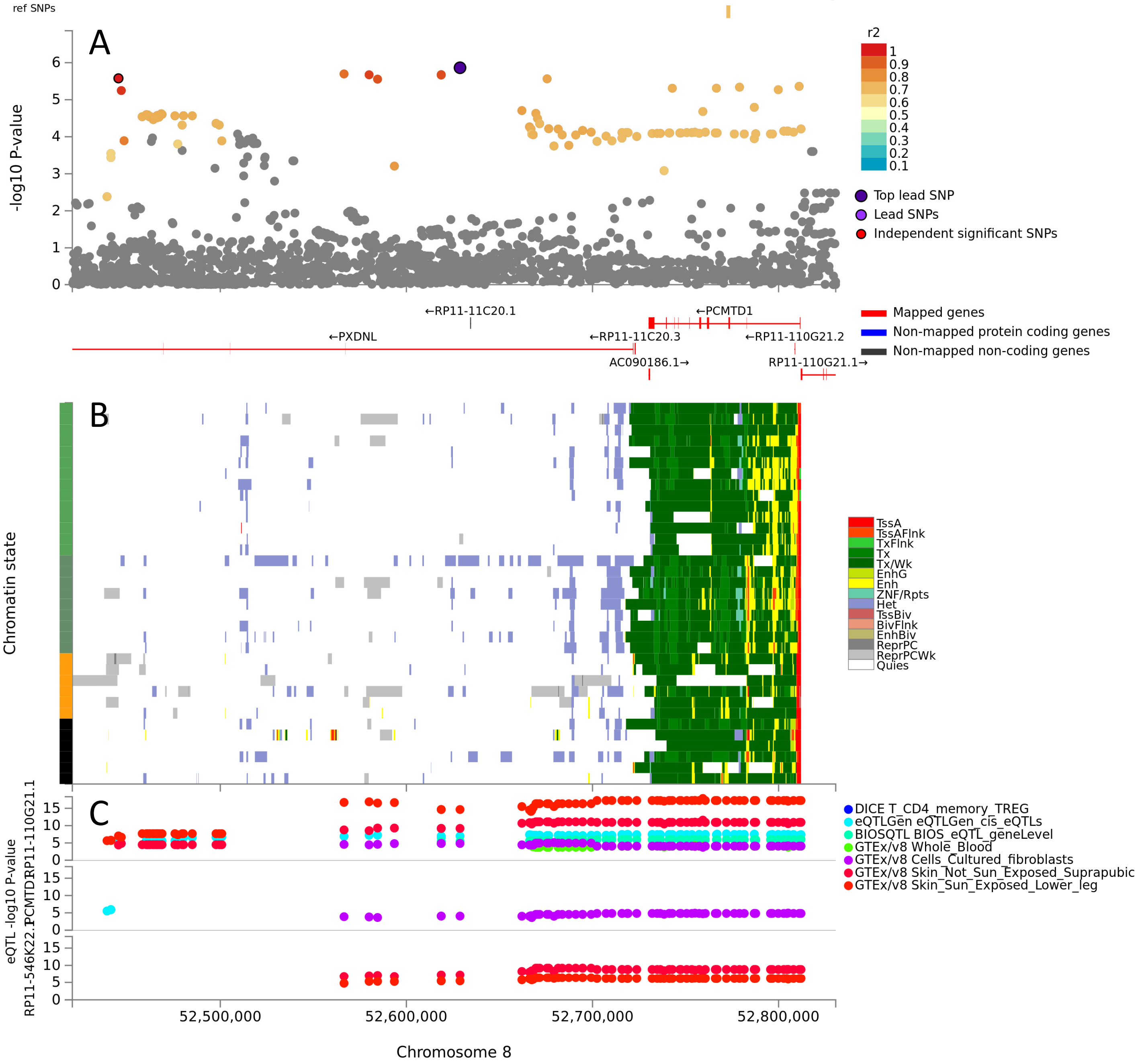

Figure S5. Results of positional, chromatin interaction, and eQTL activity mapping in FUMA for *PXDNL*/*PCMTD1* which share a common lead SNV. (A) Maps the top lead SNV, and SNVs in LD with it according to the r^2 colour-coded key, across the two genes. There was one additional independent significant SNV. (B) Chromatin-15 states colour coded for transcriptional/enhancer activity as shown in the key. Y-axis colour coding relates to cell/tissue types in which chromatin interaction was mapped. (C) eQTL activity for genes (Y-axis) in different cells/tissues from public domain databases as shown in the key. Full explanation of keys provided on introductory page for supplementary figures.

Supplementary Figure S6

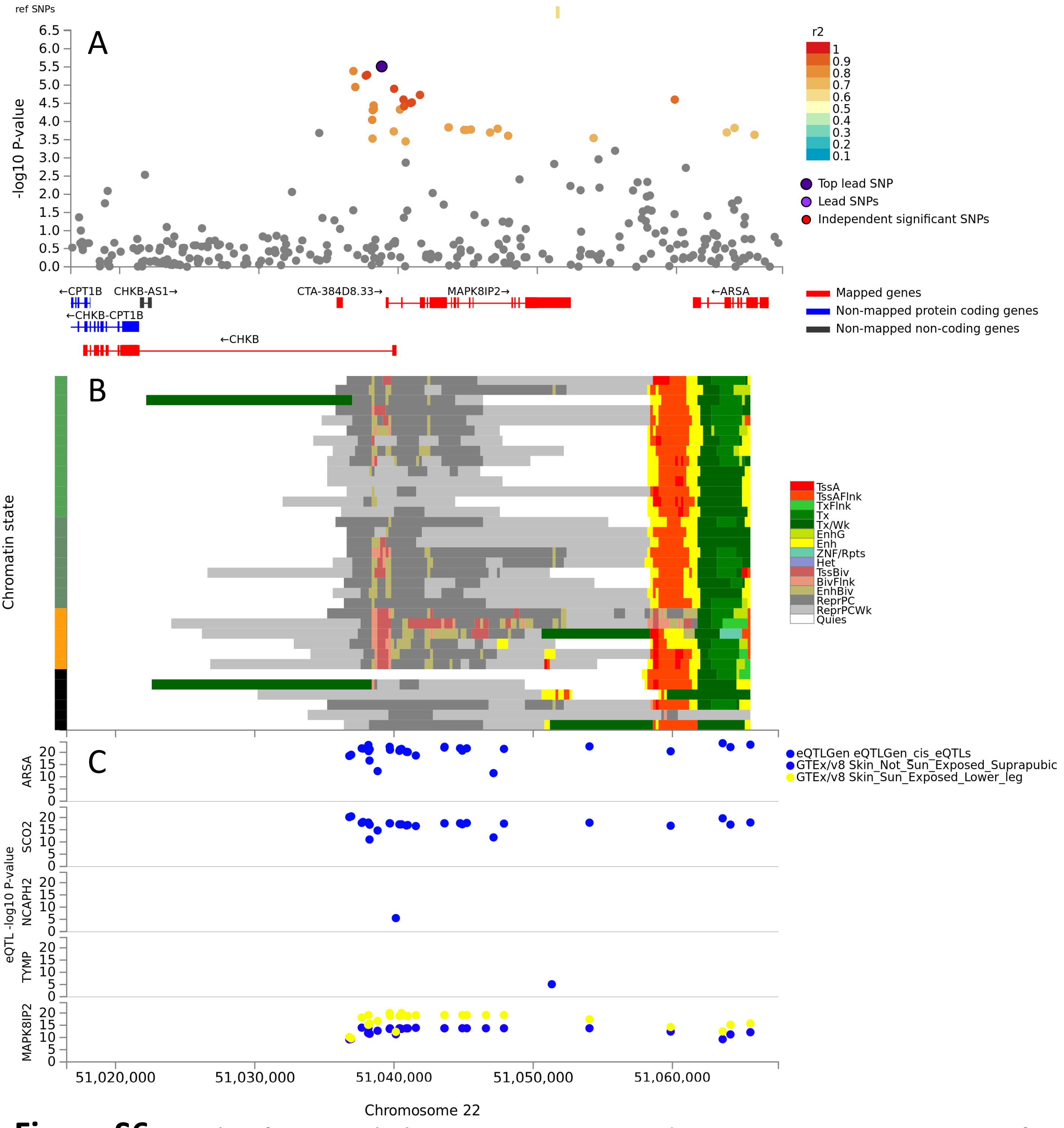

Figure S6. Results of positional, chromatin interaction, and eQTL activity mapping in FUMA for *CHKB/MAPK8IP2* which share a common lead SNV. (A) Maps the top lead SNV, and SNVs in LD with it according to the r^2 colour-coded key, across the two genes. There were no additional independent significant SNVs. (B) Chromatin-15 states colour coded for transcriptional/enhancer activity as shown in the key. Y-axis colour coding relates to cell/tissue types in which chromatin interaction was mapped. (C) eQTL activity for genes (Y-axis) in different cells/tissues from public domain databases as shown in the key. Full explanation of keys provided on introductory page for supplementary figures.

Supplementary Figure S7

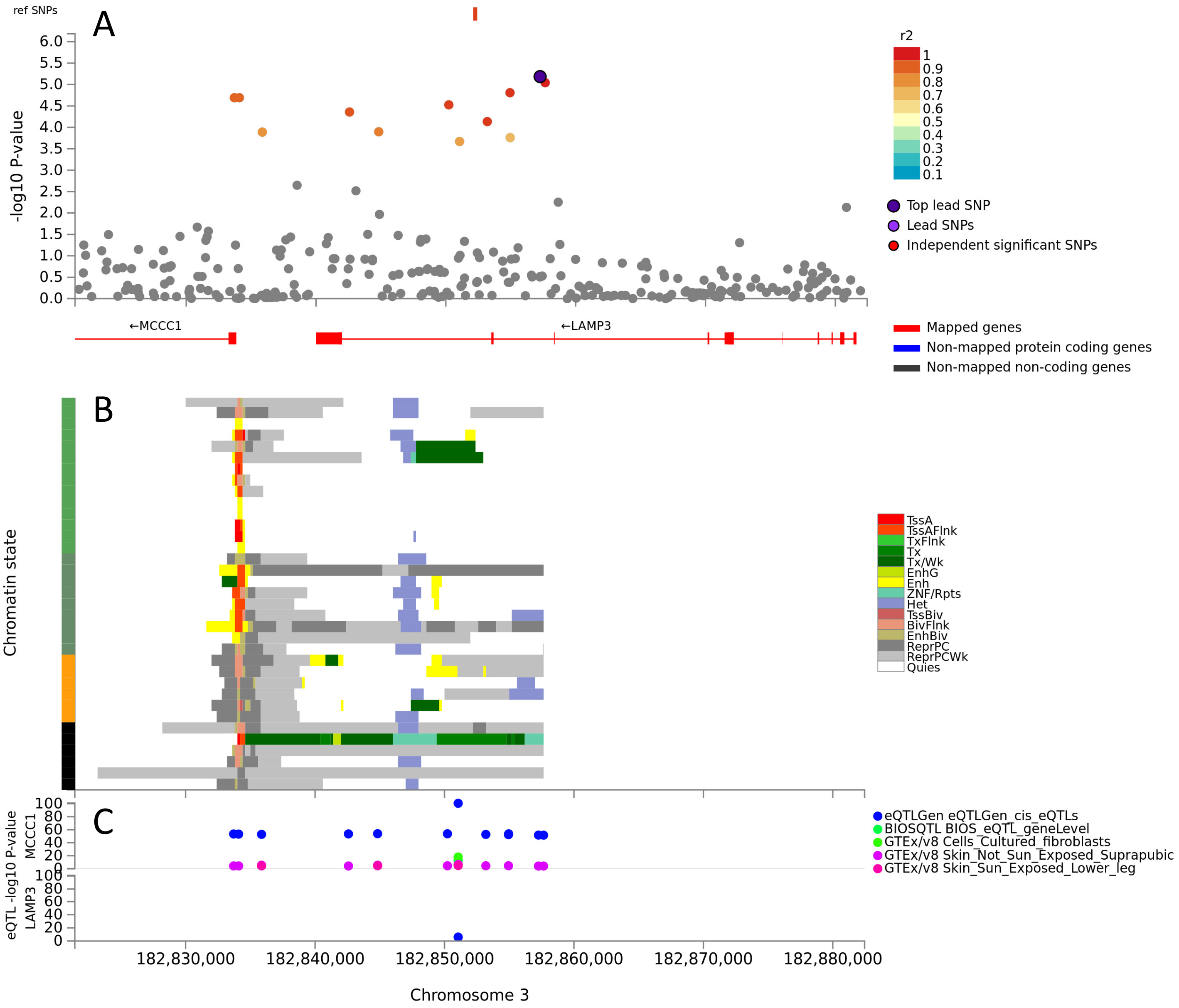

Figure S7. Results of positional, chromatin interaction, and eQTL activity mapping in FUMA for *MCCC1/LAMP3* which share a common lead SNV. (A) Maps the top lead SNV, and SNVs in LD with it according to the r^2 colour-coded key, across the two genes. There were no additional independent significant SNV. (B) Chromatin-15 states colour coded for transcriptional/enhancer activity as shown in the key. Y-axis colour coding relates to cell/tissue types in which chromatin interaction was mapped. (C) eQTL activity for genes (Y-axis) in different cells/tissues from public domain databases as shown in the key. Full explanation of keys provided on introductory page for supplementary figures.

Supplementary Figure S8

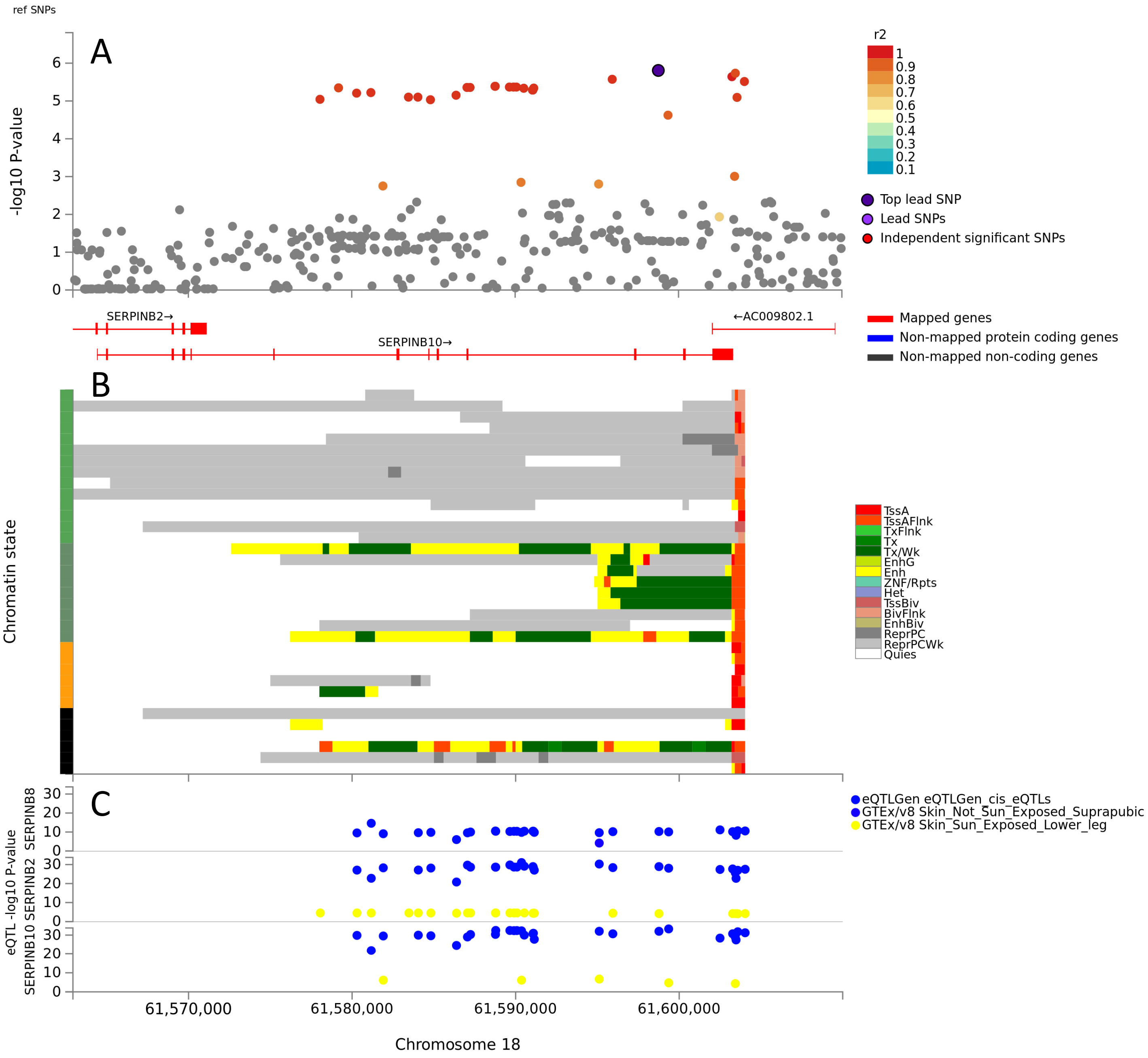

Figure S8. Results of positional, chromatin interaction, and eQTL activity mapping in FUMA for *SERPINB10*. (A) Maps the top lead SNV, and SNVs in LD with it according to the r^2 colour-coded key, across the two genes. There were no additional independent significant SNV. (B) Chromatin-15 states colour coded for transcriptional/enhancer activity as shown in the key. Y-axis colour coding relates to cell/tissue types in which chromatin interaction was mapped. (C) eQTL activity for genes (Y-axis) in different cells/tissues from public domain databases as shown in the key. Full explanation of keys provided on introductory page for supplementary figures.
