# Supplementary material for: A Genome-wide Association Study Identifies SERPINB10, CRLF3, STX7, LAMP3, IFNG-AS1, and KRT80 As Risk Loci Contributing to Cutaneous Leishmaniasis in Brazil
Source: Clin Infect Dis. 2020 Aug 23;72(10):e515–25. doi: 10.1093/cid/ciaa1230 (PMC8130031; doi:10.1093/cid/ciaa1230)
Supplement: ciaa1230_suppl_Supplementary_Tables [file ciaa1230_suppl_supplementary_tables.pdf]

Table S1. Characteristics of the Phase 1 and Phase 2 Samples.

	Phase 1			Phase 2		
	CL cases	Endemic Controls	Blood Bank Controls	CL cases	Endemic Controls	Blood Bank Controls
**N° participants[1]**						
All	956	237	631	1110	604	574
Males	560	89	420	659	273	346
Females	396	148	211	451	331	228
**Age at collection**						
Male Mean±SD	29±16	24±19	35±11	31±16	18±13	37±10
Male Range	1-78	2-81	17-65	5-87	2-75	16-65
Female Mean±SD	28±15	30±19	32±10	32±16	22±15	35±10
Female Range	4-75	1-88	16-68	3-85	2-72	17-65
**Number of lesions**						
Mean±SD	1.5±1.1	N/A	N/A	1.6±1.2	N/A	N/A
Range	1-8	N/A	N/A	1-8	N/A	N/A
N lesions females						
Mean±SD	1.6±1.1	N/A	N/A	1.6±1.2	N/A	N/A
Range[2]	1-7	N/A	N/A	1-8	N/A	N/A
N lesions males						
Mean±SD	1.5±0.9	N/A	N/A	1.6±1.1	N/A	N/A
Range	1-8	N/A	N/A	1-8	N/A	N/A
**Lesion size[3] (mm)**	(N=739)			(N=1024)		
Mean±SD	15.3±10.9	N/A	N/A	14.2±9.6	N/A	N/A
Range	1-95	N/A	N/A	1-100.5	N/A	N/A
Lesion size females	(N=284)			(N=407)		
Mean±SD	13.7±8.8	N/A	N/A	13.9±9.9	N/A	N/A
Range	2-75	N/A	N/A	2-72	N/A	N/A
Lesion size males	(N=455)			(N=617)		
Mean±SD	16.3±11.9	N/A	N/A	14.4±9.6	N/A	N/A
Range	1-95	N/A	N/A	1-100.5	N/A	N/A
**DTH (mm)**	(N=809)			(N=1091)		
Mean±SD	16.0±5.5	N/A	N/A	15.2±3.9	N/A	N/A
Range	5-46.5	N/A	N/A	4.5-37.5	N/A	N/A
Percent negative	5.9%			3.8%		
DTH (mm) females	(N=313)			(N=436)		
Mean±SD	16.3±5.6	N/A	N/A	15.5±3.8	N/A	N/A
Range	5-46.5	N/A	N/A	4.5-32.5	N/A	N/A
Percent negative	3.8%			3.8%		
DTH (mm) males	(N=496)			(N=655)		
Mean±SD	15.8±5.4	N/A	N/A	15.1±4.1	N/A	N/A
Range	5-43	N/A	N/A	4.5-37.5	N/A	N/A
Percent negative	7.2%			3.8%		

[1] Number of post-QC GWAS participants; Clinical trait measurements not available for all participants, N shown in brackets as appropriate.

[2] One female included in the GWAS had 13 lesions

[3] Diameter (mm) of largest lesion

Table S2. Details of SNP2GENE output from FUMA showing variants mapped at 32 genomic loci based on input of summary data from the combined CL GWAS.

Genomic Locus	rsID	Chr	Non Effect Allele	Effect Allele	MAF	GWAS P-value	Odds Ratio	r2	Independent Significant SNPs	Nearest Gene	Type of Gene	Distance from Nearest Gene	Functional Location	CADD Score	RDB Score	Min Chr State	Common Chr State	Pos Map Filt	Eqtl Map Filt	CI Map Filt
1	1:175280806	1	C	T	0.03	3.59E-06	1.21	1.00	1:175280806	RP3-518E13.2:TNR	antisense:protein coding	0	ncRNA_intronic	0.75	7	5	15	1	1	0
2	1:238427203	1	T	C	0.05	7.48E-06	1.10	1.00	1:238427203	RP11-136B18.1	lincRNA	4590	intergenic	0.99	6	5	15	0	0	0
3	2:24435599	2	A	G	0.01	3.56E-03	0.91	0.96	2:24599710	ITSN2		0	exonic	32.00	5	4	4	1	1	0
3	2:24599710	2	G	A	0.01	3.89E-06	0.83	1.00	2:24599710	ITSN2	protein coding	16126	intergenic	2.72	6	5	15	0	1	0
4	rs6726029	2	T	C	0.45	NA	NA	0.68	2:50706764	NRXN1		0	intronic	4.19	3a	5	15	1	0	0
4	2:50706764	2	C	CAATA	0.49	5.18E-06	0.95	1.00	2:50706764	NRXN1	protein coding	0	intronic	2.55	NA	5	15	1	0	0
5	3:22117736	3	T	A	0.04	2.18E-06	0.85	1.00	3:22117736	ZNF385D	protein coding	0	intronic	1.31	6	14	15	1	0	0
6	3:149314267	3	A	G	0.00	4.42E-06	1.20	1.00	3:149314267	WWTR1	protein coding	0	intronic	1.83	NA	2	7	1	0	0
7	3:182834095	3	T	C	0.06	2.04E-05	0.88	0.89	3:182857261	MCCC1	protein coding	231	upstream	0.51	2b	1	11	1	1	0
7	3:182857261	3	T	C	0.06	6.54E-06	0.87	1.00	3:182857261	LAMP3	protein coding	0	intronic	6.26	7	5	15	1	1	0
8	4:163402526	4	C	T	0.04	1.75E-06	0.91	1.00	4:163402526	TOMM22P4	pseudogene	40307	intergenic	0.68	5	15	15	0	0	0
9	6:132815562	6	A	C	0.01	6.10E-06	0.82	1.00	6:132815562	STX7	protein coding	0	intronic	0.99	6	2	5	1	0	0
9	6:132821875	6	C	T	0.01	8.48E-06	0.82	1.00	6:132821875	STX7	protein coding	0	intronic	1.47	6	3	5	1	0	0
9	6:132833911	6	T	C	0.01	1.20E-05	0.82	0.78	6:132845500	STX7	protein coding	0	intronic	5.39	2b	1	1	1	0	0
10	6:153979896	6	AC	A	0.02	1.73E-06	1.17	1.00	6:153979896	RP11-331O9.1		6945	intergenic	12.16	NA	5	15	0	0	0
11	7:53653377	7	T	C	0.04	1.94E-06	0.86	1.00	7:53653377	GS1-278J22.2	lincRNA	17434	intergenic	2.78	7	9	15	0	0	0
12	7:93065079	7	T	C	0.03	3.40E-06	1.16	1.00	7:93065079	CALCR	protein coding	0	intronic	5.24	5	4	15	1	0	0
13	8:40237646	8	T	C	0.16	5.04E-06	0.94	0.83	8:40245200	CTA-392C11.2		0	ncRNA_intronic	1.85	3a	5	15	1	1	0
13	8:40245200	8	G	A	0.14	1.24E-06	0.94	1.00	8:40245200	CTA-392C11.2	lincRNA	0	ncRNA_intronic	1.48	7	5	15	1	1	0
13	8:40248594	8	C	T	0.37	4.27E-03	0.97	0.66	8:40257956	CTA-392C11.2		0	ncRNA_intronic	5.83	2b	5	15	1	1	0
13	8:40254456	8	G	A	0.37	1.85E-03	0.97	0.67	8:40257956	CTA-392C11.2	lincRNA	0	ncRNA_intronic	6.02	6	5	15	1	1	0
14	8:52445279	8	C	T	0.07	2.65E-06	0.88	1.00	8:52445279	PXDNL	protein coding	0	intronic	2.85	7	5	15	1	1	0
14	8:52628820	8	A	G	0.04	1.37E-06	0.87	1.00	8:52628820	PXDNL	protein coding	0	intronic	2.92	4	5	15	1	1	0
14	8:52779001	8	C	T	0.04	4.59E-06	0.87	0.71	8:52628820	PCMTD1	protein coding	0	intronic	0.68	3a	4	5	1	1	0
14	8:52796677	8	G	T	0.04	8.42E-05	0.90	0.69	8:52628820	PCMTD1	protein coding	0	intronic	5.00	3a	2	5	1	1	0
14	8:52811035	8	T	C	0.04	4.37E-06	0.87	0.71	8:52628820	PCMTD1	protein coding	0	intronic	10.40	3a	1	1	1	1	0
15	9:34358482	9	C	A	0.07	8.07E-06	1.24	1.00	9:34358482	KIAA1161	protein coding	8185	intergenic	0.98	5	5	15	1	1	0
16	11:80470102	11	A	AAAG	0.03	4.38E-06	1.16	1.00	11:80470102	RP11-686G23.2	lincRNA	0	ncRNA_intronic	1.65	NA	7	15	1	0	0
17	11:113442125	11	A	G	0.03	7.46E-06	1.16	1.00	11:113442125	DRD2		95711	intergenic	0.07	5	5	15	0	0	0
18	12:3397404	12	G	A	0.01	2.15E-06	1.28	1.00	12:3397404	TSPAN9	protein coding	1673	intergenic	1.63	5	4	15	1	0	0
18	rs116462346	12	G	A	0.01	NA	NA	0.84	12:3397404	RP5-1063M23.1	lincRNA	301	downstream	2.04	2b	5	14	1	0	0
18	12:3417881	12	C	G	0.01	1.46E-05	1.23	0.61	12:3397404	RP5-1063M23.1	lincRNA	8490	intergenic	3.09	2b	5	14	1	0	0
19	rs7978828	12	A	T	0.15	NA	NA	0.61	12:52590004	KRT80	protein coding	0	intronic	1.23	3a	1	7	1	1	0
19	12:52589258	12	T	C	0.19	3.17E-05	1.06	0.90	12:52590004	KRT80	protein coding	3473	intergenic	13.19	3a	5	7	1	1	0
19	12:52590004	12	A	G	0.21	6.58E-06	1.06	1.00	12:52590004	KRT80	protein coding	4219	intergenic	7.84	6	5	5	1	1	0
19	12:52591689	12	T	G	0.15	2.85E-03	1.05	0.65	12:52590004	KRT80	protein coding	5904	intergenic	0.67	3a	2	7	1	1	0
20	12:68401391 **	12	T	A	0.28	7.24E-05	1.05	0.95	12:68407845	IFNG-AS1	antisense	0	ncRNA_intronic	2.53	3a	5	15	1	1	0
20	12:68407845 **	12	G	C	0.29	1.32E-05	1.06	1.00	12:68407845	IFNG-AS1	antisense	0	ncRNA_intronic	1.73	7	5	15	1	1	0
21	13:32209771	13	G	C	0.11	5.00E-06	1.07	1.00	13:32209771	RXFP2		103902	intergenic	1.82	7	5	15	0	0	0
22	14:47560881	14	C	T	0.01	9.52E-07	1.28	1.00	14:47560881	MDGA2:MDGA2	protein coding	00:00	intronic	0.36	6	5	15	1	0	0
23	14:53686046	14	T	C	0.01	2.39E-06	0.84	1.00	14:53686046	AL163953.3	lincRNA	0	ncRNA_intronic	6.44	NA	1	15	1	0	0
23	14:53786705	14	C	G	0.01	3.08E-06	0.83	1.00	14:53786705	AL163953.3	lincRNA	0	ncRNA_intronic	1.88	7	5	15	1	0	0
24	16:26466019	16	T	C	0.07	8.61E-06	1.10	1.00	16:26466019	AC130464.1		96342	intergenic	7.58	6	5	15	0	0	0
25	17:29136126	17	T	C	0.01	5.12E-06	0.83	1.00	17:29136126	CRLF3	protein coding	0	intronic	14.53	4	1	5	1	0	0
26	18:46766154	18	A	G	0.14	2.18E-06	1.09	1.00	18:46766154	DYM	protein coding	0	intronic	2.30	5	4	5	1	1	0
26	18:46801104	18	C	T	0.12	2.07E-02	1.05	0.78	18:46766154	DYM	protein coding	0	intronic	6.37	2b	4	5	1	1	0
26	18:46888869	18	A	G	0.14	1.70E-03	1.06	0.91	18:46766154	DYM	protein coding	0	intronic	13.11	3a	4	5	1	1	0
26	18:46925522	18	T	A	0.14	3.24E-03	1.07	0.86	18:46766154	DYM	protein coding	0	intronic	4.00	2b	2	7	1	1	0
26	18:46928004	18	A	T	0.14	2.44E-03	1.07	0.86	18:46766154	DYM	protein coding	0	intronic	0.00	3a	5	15	1	0	0
26	18:46928006	18	A	T	0.14	2.02E-03	1.07	0.87	18:46766154	DYM	protein coding	0	intronic	0.01	3a	5	15	1	0	0
26	18:46928067	18	A	C	0.14	2.88E-03	1.07	0.86	18:46766154	DYM	protein coding	0	intronic	0.83	3a	5	15	1	1	0
26	rs185458861	18	C	G	0.16	NA	NA	0.67	18:46766154	DYM	protein coding	0	intronic	5.19	2b	1	1	1	1	0
26	rs75077262	18	G	A	0.14	NA	NA	0.85	18:46766154	DYM	protein coding	0	intronic	8.22	2b	1	1	1	1	0
26	rs78378550	18	C	G	0.16	NA	NA	0.68	18:46766154	DYM	protein coding	0	intronic	6.46	2b	1	1	1	1	0
26	18:46987023	18	G	C	0.12	2.66E-02	1.06	0.76	18:46766154	DYM	protein coding	0	UTR5	10.50	3a	1	1	1	1	0
26	rs78131007	18	G	A	0.16	NA	NA	0.67	18:46766154	DYM	protein coding	199	upstream	5.38	2b	1	15	1	1	0
27	18:52935901	18	G	C	0.06	1.89E-03	0.94	0.99	18:52955675	TCF4	protein coding	0	intronic	12.32	3a	3	4	1	0	0
27	18:52955675	18	T	C	0.06	5.70E-06	0.91	1.00	18:52955675	TCF4	protein coding	0	intronic	0.64	5	5	15	1	0	0
28	18:61587053	18	A	G	0.17	4.39E-06	1.06	0.97	18:61598763	SERPINB10	protein coding	0	exonic	18.22	7	7	15	1	1	0
28	18:61598763	18	C	T	0.18	1.56E-06	1.07	1.00	18:61598763	SERPINB10	protein coding	0	intronic	5.20	6	5	15	1	1	0
28	18:61603476	18	T	G	0.17	1.84E-06	1.07	0.94	18:61598763	AC009802.1	protein coding	0	intronic	2.32	2b	1	1	1	1	0
28	18:61603581	18	A	T	0.17	8.04E-06	1.06	0.96	18:61598763	AC009802.1	protein coding	0	intronic	8.63	2a	1	1	1	1	0
28	18:61604032	18	T	G	0.17	3.06E-06	1.07	0.97	18:61598763	AC009802.1	protein coding	0	intronic	6.93	3a	1	1	1	1	0
29	19:55738634	19	T	C	0.08	2.87E-04	0.90	0.68	19:55746886	TMEM86B:AC010327.	miRNA	0	exonic	11.45	2b	3	4	1	1	0
29	19:55746886	19	C	T	0.08	4.18E-06	0.84	1.00	19:55746886	PPP6R1	protein coding	0	intronic	1.44	6	4	4	1	1	0
29	19:55752046	19	C	T	0.08	7.25E-06	0.84	0.96	19:55746886	PPP6R1	protein coding	0	intronic	0.15	2b	3	4	1	1	0
29	19:55771447	19	T	A	0.09	2.38E-04	0.88	0.69	19:55746886	PPP6R1	protein coding	1083	intergenic	4.48	2b	1	7	1	1	0
30	21:24193385	21	A	G	0.27	6.47E-06	1.06	1.00	21:24193385	AP000949.1		61517	intergenic	2.95	NA	9	15	0	0	0
30	21:24199888	21	C	T	0.27	2.26E-05	1.05	1.00	21:24193385	AP000949.1		55014	intergenic	1.12	3a	9	15	0	0	0
31	rs2070429	21	G	A	0.27	NA	NA	0.69	21:48023640	DIP2A	protein coding	0	intronic	8.14	1f	2	4	1	1	0
31	21:48023640	21	T	*TAGAT(	0.26	9.31E-06	1.06	1.00	21:48023640	S100B	protein coding	0	intronic	4.04	NA	1	15	1	1	0
31	21:48025097	21	A	G	0.27	6.77E-05	1.05	0.88	21:48023640	S100B	protein coding	0	UTR5	10.02	2b	1	7	1	1	0
31	21:48027177	21	G	A	0.32	1.69E-05	1.05	0.65	21:48023640	S100B	protein coding	2055	intergenic	1.34	1f	2	15	1	1	0
31	rs993461	21	G	T	0.30	NA	NA	0.64	21:48023640	S100B	protein coding	12221	intergenic	10.13	2b	5	15	0	1	0
32	22:51038824	22	G	C	0.06	3.07E-06	1.09	1.00	22:51038824	CHKB	protein coding	0	upstream	11.35	4	1	13	1	1	0
32	22:51040387	22	C	G	0.07	2.49E-05	1.07	0.94	22:51038824	MAPK8IP2	protein coding	0	intronic	0.09	2b	1	13	1	1	0
32	22:51040537	22	T	C	0.05	3.52E-04	1.07	0.75	22:51038824	MAPK8IP2	protein coding	0	intronic	6.02	2b	1	13	1	1	0
32	22:51044746	22	G	C	0.05	1.71E-04	1.07	0.75	22:51038824	MAPK8IP2	protein coding	0	intronic	1.63	1f	4	13	1	1	0
32	22:51051337:A:C	22	C	A	0.05	NA	NA	0.61	22:51038824	MAPK8IP2	protein coding	0	UTR3	0.38	2b	4	14	1	1	0

* TAGATGTTAAAA
**12:68401391 rs12422954
**12:68407845 rs4913269

Key for RDB scores:

RegulomeDB Categorical Scores

Category	Description
	Likely to affect binding and linked to expression of a gene target
1a	eQTL + TF binding + matched TF motif + matched DNase footprint + DNase peak
1b	eQTL + TF binding + any motif + DNase footprint + DNase peak
1c	eQTL + TF binding + matched TF motif + DNase peak
1d	eQTL + TF binding + any motif + DNase peak
1e	eQTL + TF binding + matched TF motif
1f	eQTL + TF binding/DNase peak
	Likely to affect binding
2a	TF binding + matched TF motif + matched DNase footprint + DNase peak
2b	TF binding + any motif + DNase footprint + DNase peak
2c	TF binding + matched TF motif + DNase peak
	Less likely to affect binding
3a	TF binding + any motif + DNase peak
3b	TF binding + matched TF motif
	Minimal binding evidence
4	TF binding + DNase peak
5	TF binding or DNase peak
6	Motif hit
	No binding evidence
7	No evidence
NA	the variant does not exist in RegulomeDB

Table S3. Original GWAS data and review of gene function in relation to parasite biology and CL immunopathology for post-GWAS hits identified using SNP2GENE in FUMA and transcriptional mapping data (see Table 1, main text). Only the most significant variant is shown for each gene/genomic locus. A1 is the associated allele for the odds ratio shown. Alleles are reference/alternative. Expression data are from the project HPA RNA-seq normal tissues which undertook RNA-seq of tissue samples from 95 human individuals representing 27 different tissues in order to determine tissue-specificity of all protein-coding genes (BioProject: PRJEB4337; Publication: PMID 24309898). As functional candidates for CL we focussed on genes expressed in skin, lymph node (LN) or bone marrow (BM). Expression in CL lesions versus normal skin (in bold) is taken from Novais et al. (Publication: PMID 25036052; GEO database Accession GSE55664). Functional information is taken from NCBI's Gene Database unless otherwise indicated with a PMID reference number. Genes highlighted in bold are those functional candidates presented in more detail in the main paper.

Chr	Position (bp)	rsID	P value	Odds Ratio (SE)	Beta (SE)	A1	Consequence	Alleles	GENE	Full name	Expression	Function
3	22117736	rs1383086	2.18E-06	0.853 (0.794-0.916)	-0.034 (0.008)	T	intron	A/T	ZNF385D	Zinc finger protein 385D	Broad expression in testis, brain and 16 other tissues, including low expression in skin. **Marginally down-regulated in CL lesion versus normal skin Padj 6.28E-04, LFC -0.81 (Novais).**	Function unknown.
3	182834095	rs7649244	2.04E-05	0.88 (0.83-0.934)	-0.032 (0.008)	T	upstream	C/T	MCCC1	Methylcrotonoyl-CoA carboxylase 1	Ubiquitous expression in fat, kidney and 25 other tissues, moderate in skin, BM and LN. **Strongly down-regulated in CL lesion compared to normal skin Padj 7.98E-10, LFC -1.06 (Novais).**	Downstream of LAMP3. Large subunit of 3-methylcrotonyl-CoA carboxylase. A biotin-dependent mitochondrial enzyme that catalyzes the conversion of 3-methylcrotonyl-CoA to 3-methylglutaconyl-CoA, a critical step for leucine and isovaleric acid catabolism. Associated with Parkinson's Disease (Pubmed 23496138; 30957308) and with 3-methylcrotonyl-coenzyme A Carboxylase Deficiency in neonates (Pubmed 29978827; 27601257).
3	182857261	rs74285558	6.54E-06	0.868 (0.817-0.923)	-0.034 (0.008)	T	intron	C/T	**LAMP3**	Lysosomal associated membrane protein 3. Also known as DCLAMP.	Biased expression in lung, appendix and 3 other tissues including LN and small amount in skin. **Strongly up-regulated in CL lesion compared to normal skin Padj 9.25E-12, LFC 2.56 (Novais).**	Dendritic cells (DCs) are the most potent antigen-presenting cells. Immature DCs efficiently capture antigens and differentiate into interdigitating dendritic cells (IDCs) in lymphoid tissues that induce primary T-cell responses (summary by de Saint-Vis et al., 1998 [PubMed 9768752]).
6	132815562	rs144488134	6.10E-06	0.821 (0.754-0.894)	-0.034 (0.007)	A	intron	C/A	**STX7**	Syntaxin 7	Ubiquitous expression in lymph node, spleen and 24 other tissues including skin and BM. **Marginally up-regulated CL lesion versus normal skin Padj 0.008; LFC 0.38 (Novais).**	A syntaxin family membrane receptor involved in vesicle transport. The encoded protein binds alpha-SNAP, an important regulator of transport vesicle fusion. Along with syntaxin 13, this **protein plays a role in the ordered fusion of endosomes and lysosomes with the phagosome**.

7	93065079	rs143586968	3.40E-06	1.159 (1.089-1.234)	0.037 (0.008)	T	intron	C/T	CALCR	Calreticulin receptor	Biased expression kidney, test; absent skin, LN and BM. **Moderately upregulated in CL lesion versus normal skin Padj 3.62E-04, LFC 0.754 (Novais).**	Calcitonin receptor, a high affinity receptor for the peptide hormone calcitonin and belongs to a subfamily of seven transmembrane-spanning G protein-coupled receptors. The encoded protein is involved in maintaining calcium homeostasis and in regulating osteoclast-mediated bone resorption. Polymorphisms in this gene have been associated with variations in bone mineral density and onset of osteoporosis.
8	52628820	rs13261618	1.37E-06	0.868 (0.819-0.919)	-0.037 (0.008)	A	intron	G/A	PXDNL	Peroxidasin like	Biased expression in heart, testis, low expression in kidney and fat. **Not different in CL lesion versus normal skin (Novais).**	Peroxidasin like. Alzheimer - associated with neuritic plaques. Severe brain phenotype in KO humans PMID: 28640246.
8	52628820	rs13261618	1.37E-06	0.868 (0.819-0.919)	-0.035 (0.008)	A	downstream	G/A	PCMTD1	Protein-L-isoaspartate (D-aspartate) O-methyltransferase domain containing 1	Ubiquitous expression in thyroid, fat and 25 other tissues, including skin and lymph node. **Significantly down-regulated in CL lesion versus normal skin Padj 6.20E-09, LFC -0.961 (Novais).**	Protein-L-isoaspartate (D-aspartate) O-methyltransferase domain containing 1. Associated with Glaucoma in multiple genetic studies.
12	3397404	rs77563142	2.15E-06	1.277 (1.154-1.413)	0.035 (0.007)	G	downstream	A/G	TSPAN9	Tetraspanin 9	Broad expression in heart, placenta and 23 other tissues, including skin. Low in lymph node and BM. **Significantly down-regulated CL lesion versus normal skin Padj 2.54E-05. LFC -0.836 (Novais).**	Tetraspanin 9, member of the transmembrane 4 superfamily, also known as the tetraspanin family. Most of these members are cell-surface proteins that are characterized by the presence of four hydrophobic domains. The proteins mediate signal transduction events that play a role in the regulation of cell development, activation, growth and motility.
12	52590004	rs10783496	6.58E-06	1.059 (1.033-1.086)	0.035 (0.008)	A	upstream	G/A	**KRT80**	Keratin 80	Biased expression in skin, esophagus - not expressed in lymph node or BM. **Significantly down-regulated CL lesion versus normal skin Padj 3.07E-08, LFC -1.59 (Novais).**	Keratin 80. Keratins are intermediate filament proteins responsible for the structural integrity of epithelial cells and are subdivided into epithelial keratins and hair keratins. This gene's expression profile shows that it encodes a type II epithelial keratin, although structurally the encoded protein is more like a type II hair keratin. This protein is involved in cell differentiation, localizing near desmosomal plaques in earlier stages of differentiation but then dispersing throughout the cytoplasm in terminally differentiating cells. The type II keratins are clustered in a region of chromosome 12q13.

12	68407845	rs4913269	1.32E-05	1.057 (1.031-1.084)	0.033 (0.008)	G	intron	C/G	**IFNG-AS1**	IFNG antisense RNA 1	Biased expression in lymph node, appendix and 7 other tissues. No expression in skin or BM. **A probe for his gene is not on the chip used by Novais.**	See Stein et al (2019) in iScience (PMID:30661002): The lncRNA IFNG-AS1 was found to strongly influence the responses to several pathogens in mice by increasing interferon gamma (IFNg) secretion. Studies have looked at IFNG-AS1 in T cells, yet IFNG-AS1 function in natural killer cells (NKs), an important source of IFNg, remains unknown. Here, we show a previously undescribed sequence of IFNG-AS1 and report that it may be more abundant in cells than previously thought. Using primary human NKs and an NK line with IFNG-AS1 overexpression, we show that IFNG-AS1 is quickly induced upon NK cell activation, and that IFNG-AS1 overexpression leads to increased IFNg secretion. Taken together, our work expands IFNG-AS1's activity to the innate arm of the type I immune response.
17	29136126	rs75270613	5.12E-06	0.832 (0.769-0.901)	-0.034 (0.008)	T	intron	C/T	**CRLF3**	Cytokine receptor like factor 3	Broad expression in LN, appendix and 24 other tissues, including skin and BM. **Significantly upregulated in CL lesions compared to normal skin Padj 9.19E-09, LFC 0.981 (Novais).**	Cytokine receptor like factor 3, a cytokine receptor-like factor that may negatively regulate cell cycle progression at the G0/G1 phase. Studies of the related rat protein suggest that it may regulate neuronal morphology and synaptic vesicle biogenesis. This gene is one of several genes located in the neurofibromatosis type I tumor suppressor region on the q arm of chromosome 17, a region that is subject to microdeletions, duplications, chromosomal breaks and rearrangements. One of 14 genes found to be dysregulated in squamuos cell carcinoma and its precursor lesion actinic keratosis.
18	46766154	rs4939853	2.18E-06	1.09 (1.052-1.13)	0.036 (0.008)	G	intron	A/G	DYM	Dymeclin	Ubiquitous expression in thyroid, ovary and 25 other tissues including skin, lymph node and BM. **Not different CL lesions versus normal skin (Novais).**	Dymeclin, a protein which regulates Golgi-associated secretory pathways that are essential to endochondral bone formation during early development. This gene is also believed to play a role in early brain development. This gene is widely expressed in embryos and is particularly abundant in chodrocytes and brain tissues. It encodes a peripheral membrane protein which shuttles between the cytosol and Golgi complex.
18	52955675	rs8090418	5.70E-06	0.909 (0.873-0.947)	-0.035 (0.008)	T	intron	C/T	TCF4	Transcription factor 4	Ubiquitous expression in brain, fat and 24 other tissues including LN and skin, lower in BM. **Not signficantly different CL lesion versus normal skin (Novais).**	Transcription factor 4, a basic helix-loop-helix transcription factor. The encoded protein recognizes an Ephrussi-box ('E-box') binding site ('CANNTG') - a motif first identified in immunoglobulin enhancers. This gene is broadly expressed, and may play an important role in nervous system development. Defects in this gene are a cause of Pitt-Hopkins syndrome.

18	61598763	rs8084306	1.56E-06	1.068 (1.04-1.097)	0.038 (0.008)	C	intron	T/C	**SERPINB10**	Serpin family B member 10	Restricted expression toward BM; absent all other tissues. **No expression CL lesions (Novais).**	Serpin family B member 10, a member of the serpin peptidase inhibitor, clade B family and is found in a cluster of other similar genes on chromosome 18. The protein encoded by this gene appears to help control the regulation of protease functions during hematopoiesis.
19	55762225	rs3848610	4.93E-06	0.836 (0.774-0.903)	-0.035 (0.008)	T	intron	C/T	PPP6R1	Protein phosphatase 6 regulatory subunit 1	Broad expression in testis, lymph node, BM and 23 other tissues; lower in skin. **Significantly upregulated in CL lesion compared to normal skin Padj 6.09E-07, LFC 0.76**7 **(Novais)**.	Protein phosphatase 6 regulatory subunit 1. Protein phosphatase regulatory subunits, such as SAPS1 (=PPP6R1), modulate the activity of protein phosphatase catalytic subunits by restricting substrate specificity, recruiting substrates, and determining the intracellular localization of the holoenzyme. SAPS1 is a regulatory subunit for the protein phosphatase-6 catalytic subunit (PPP6C; MIM 612725) (Stefansson and Brautigan, 2006 [PubMed 16769727]).
21	48023640	rs201555201	9.31E-06	1.061 (1.033 - 1.089)	0.059(0.013).	TAGATGTTAAAA	intronic	TAGATGT TAAAA/T	S100B	S100 calcium binding protein B	Biased expression in brain, fat and 2 other tissues; absent skin, LN and BM. **Not different CL lesions versus normal skin (Novais).**	The protein encoded by this gene is a member of the S100 family of proteins containing 2 EF-hand calcium-binding motifs. S100 proteins are localized in the cytoplasm and/or nucleus of a wide range of cells, and involved in the regulation of a number of cellular processes such as cell cycle progression and differentiation.
22	51038824	rs1239627205	3.07E-06	1.086 (1.049-1.124)	0.036 (0.008)	G	upstream downstream	C/G	MAPK8IP2 CHKB	CHKB choline kinase beta	Ubiquitous expression in spleen (RPKM 35.8), lymph node (RPKM 28.9) and 25 other tissues, including skin and BM. **Upregulated CL lesion versus normal skin Padj 2.60E-05, LFC 0.496 (Novais).**	CHKB choline kinase beta, Choline kinase (CK) and ethanolamine kinase (EK) catalyze the phosphorylation of choline/ethanolamine to phosphocholine/phosphoethanolamine. This is the first enzyme in the biosynthesis of phosphatidylcholine/phosphatidylethanolamine in all animal cells. The highly purified CKs from mammalian sources and their recombinant gene products have been shown to have EK activity also, indicating that both activities reside on the same protein. The choline kinase-like protein encoded by CHKL belongs to the choline/ethanolamine kinase family; however, its exact function is not known.
										MAPK8IP2 mitogen-activated protein kinase 8 interacting protein 2	Biased expression in brain (RPKM 21.8), adrenal (RPKM 2.8) and 2 other tissues, but NOT skin, BM or lymph nodes. **Not different CL lesion versus normal skin (Novais).**	MAPK8IP2 mitogen-activated protein kinase 8 interacting protein 2, a scaffold protein that is thought to be involved in the regulation of the c-Jun amino-terminal kinase signaling pathway. This protein has been shown to interact with and regulate the activity of MAPK8/JNK1 and MAP2K7/MKK7 kinases.
