# Supplementary material for: A Genome-wide Association Study Identifies SERPINB10, CRLF3, STX7, LAMP3, IFNG-AS1, and KRT80 As Risk Loci Contributing to Cutaneous Leishmaniasis in Brazil
Source: Clin Infect Dis. 2020 Aug 23;72(10):e515–25. doi: 10.1093/cid/ciaa1230 (PMC8130031; doi:10.1093/cid/ciaa1230)
Supplement: ciaa1230_suppl_Supplementary_Information [file ciaa1230_suppl_supplementary_information.pdf]

A Genome-Wide Association Study Highlights a Regulatory Role for *IFNG-AS1* Contributing to Cutaneous Leishmaniasis In Brazil

Léa C. Castellucci,[1,2,*] Lucas Almeida,[1,2,*] Svetlana Cherlin,[3,*] Michaela Fakiola,[4,*,†] Richard W. Francis,[5] Edgar M. Carvalho,[1] Anadílton Santos da Hora,[2] Tainã Souza do Lago,[2] Amanda B. Figueiredo,[6] Clara M. Cavalcanti,[6] Natalia S. Alves,[6] Katia LP Morais.[6] Andréa Teixeira-Carvalho,[7] Walderez O. Dutra,[1,8] Kenneth J. Gollob,[1,6,9] Heather J. Cordell,[3] and Jenefer M. Blackwell[5,10] [*]Contributed equally

[1]National Institute of Science and Technology in Tropical Diseases, Brazil; [2]Federal University of Bahia, Salvador, Brazil; [3]Population Health Sciences Institute, Newcastle University, UK; [4]INGM-National Institute of Molecular Genetics "Romeo ed Enrica Invernizzi" Milan, Milan, Italy; [5]Telethon Kids Institute, The University of Western Australia, Western Australia; [6]International Center for Research, AC Camargo Cancer Center, São Paulo, Brazil; [7]Instituto Rene Rachou of Fundação Oswaldo Cruz (FIOCRUZ-Minas), Belo Horizonte, Brazil; [8]Instituto de Ciências Biológicas, Universidade Federal de Minas Gerais, Belo Horizonte, Brazil; [9]Núcleo de Ensino e Pesquisa, Instituto Mario Penna, Belo Horizonte, Brazil; [10]Department of Pathology, University of Cambridge, UK; [†]Present address: IFOM, the FIRC Institute of Molecular Oncology, Milan, Italy

Corresponding authors: Jenefer M. Blackwell (jenefer.blackwell@telethonkids.org.au) and Kenneth Gollob (kenneth.gollob@accamargo.org.br)

SUPPLEMENTARY INFORMATION

Candidate gene studies [1-9] of *L. braziliensis* suggest multiple genes associated with pro- and anti-inflammatory responses (*TNFA*, *SLC11A1*, *CXCR1*, *IL6*, *IL10, CCL2/MCP1, IFNG*) and/or wound healing (*FLI1*, *CTGF*, *TGFBR2*, *SMAD2*, *SMAD3*, *SMAD7*, *COL1A1*) influence CL or ML disease. Although frequently underpinned by functional data [5-7, 9] and/or supported by prior immunological studies [10-12], these studies have generally lacked statistical power. Since it is customary in GWAS to examine the data for evidence of associations identified in published candidate gene studies, we present this analysis here as supplementary information.

Interrogating the GWAS Data in Relation to Previous Candidate Gene Studies

We interrogated the GWAS data to determine whether there was support for candidacy of previously studied genes [1-9]. Supplementary Information Table 1 presents the top hits for SNVs within previous candidate, or functionally related, genes. Given *a priori* evidence to examine these genes, we used P<0.01 as cut-off. No variants were associated at P<0.01 for *TNFA*, *SLC11A1*, *CXCR1*, *IL6*, *IL10*, *CCL2/MCP1, IFNG, FLI1*, *CTGF*, *COL1A1*, or *TGFBR2*. Associations were observed at *SMAD2*, *SMAD3* and *SMAD7*, wound healing genes previously associated with CL [2]. The strongest signal was at *SMAD2* (Supplementary Information Figure 1A); *SMAD3* and *SMAD7* (Supplementary Information Figure 1A also includes *SMAD7*) were less well-supported. Of note, for these genes the specific SNVs used in candidate gene studies were not present on the chips employed, or in the imputed data. Therefore, the LocusZoom plots (Supplementary Information Figure 1) are provided to demonstrate the extent of association across the genes of interest. Associations were observed at related SMADs: *SMAD1*, *SMAD4* (also in Supplementary Information Figure 1A), *SMAD6* and *SMAD9*. SMADs transduce signals from transforming growth factor beta (TGFβ) receptors. Although no association at P<0.01 was observed at *TGFBR2*, association at the functionally related gene *TGFBR3* (Supplementary Information Figure 1B) was supported at multiple variants. Similarly, associations observed at collagen genes, *COL24A1* (Supplementary Information Figure 1C) and *COL11A1* (Supplementary Information Figure 1D), functionally related to wound healing gene *COL1A1* were supported at multiple variants across each gene. Associations were observed for variants at genes (*IL6R* and *IL10R*) encoding receptors for cytokines IL-6/IL-10 shown to be genetically and/or functionally associated with cutaneous forms of *L. braziliensis* disease [5, 6].

Discussion of candidate gene analysis

In relation to previous candidate gene studies, a role for wound healing genes in CL [2] was supported here by associations at SMAD genes, the strongest for variants at *SMAD2*. SMAD proteins transduce signals from receptors of the TGFβ superfamily. Although we did not replicate the earlier [2] genetic association with *TGFBR2* encoding the type II TGFβ receptor, we did see association at *TGFBR3* encoding the type III receptor. TGFBR3 is a membrane proteoglycan [13] that often functions as a co-receptor with other TGFβ receptors [14]. Ectodomain shedding produces soluble TGFBR3 which inhibits TGFβ signalling [13]. TGFβ plays an important role in tissue fibrosis [15], with abnormal TGFβ function implicated in a large number (reviewed [16]) of fibrotic and inflammatory pathologies. CL lesions comprise a combination of inflammatory processes and fibrosis [17]. Fibrotic reaction leads to production of extracellular matrix proteins including collagens, and to activation of local fibroblasts to differentiate into myofibroblasts. TGFβ stimulates COL11A1 expression in dermal fibroblasts [18], while novel myofibroblast-specific expression of COL24A1 is a signature of skin wounds [19]. Together with associations at *IL6R* and *IL10R*, our analysis of previous candidate genes in relation to GWAS data provides modest support for the complex interplay between fibrotic and inflammatory processes in pathologies associated with CL caused by *L. braziliensis.*

References

1. Cabrera M, Shaw M-A, Sharples C, et al. Polymorphism in TNF genes associated with mucocutaneous leishmaniasis. JExpMed **1995**; 182:1259-64.
2. Castellucci L, Jamieson SE, Almeida L, et al. Wound healing genes and susceptibility to cutaneous leishmaniasis in Brazil. Infect Genet Evol **2012**; 12:1102-10.
3. Castellucci L, Jamieson SE, Miller EN, et al. FLI1 polymorphism affects susceptibility to cutaneous leishmaniasis in Brazil. Genes Immun **2011**; 12:589-94.
4. Castellucci L, Jamieson SE, Miller EN, et al. CXCR1 and SLC11A1 polymorphisms affect susceptibility to cutaneous leishmaniasis in Brazil: a case-control and family-based study. BMC Med Genet **2010**; 11:10.
5. Castellucci L, Menezes E, Oliveira J, et al. IL6 -174 G/C promoter polymorphism influences susceptibility to mucosal but not localized cutaneous leishmaniasis in Brazil. J Infect Dis **2006**; 194:519-27.
6. Salhi A, Rodrigues V, Jr., Santoro F, et al. Immunological and genetic evidence for a crucial role of IL-10 in cutaneous lesions in humans infected with Leishmania braziliensis. J Immunol **2008**; 180:6139-48.
7. Ramasawmy R, Menezes E, Magalhaes A, et al. The -2518bp promoter polymorphism at CCL2/MCP1 influences susceptibility to mucosal but not localized cutaneous leishmaniasis in Brazil. Infect Genet Evol **2010**; 10:607-13.
8. Almeida L, Oliveira J, Guimaraes LH, Carvalho EM, Blackwell JM, Castellucci L. Wound healing genes and susceptibility to cutaneous leishmaniasis in Brazil: role of COL1A1. Infect Genet Evol **2015**; 30:225-9.
9. da Silva GAV, Mesquita TG, Souza VC, et al. A Single Haplotype of IFNG Correlating With Low Circulating Levels of Interferon-gamma Is Associated With Susceptibility to Cutaneous Leishmaniasis Caused by Leishmania guyanensis. Clin Infect Dis **2019**.
10. Castes M, Trujillo D, Rojas ME, et al. Serum levels of tumor necrosis factor in patients with American cutaneous leishmaniasis. Biol Res **1993**; 26:233-8.
11. D'Oliveira A, Jr., Machado P, Bacellar O, Cheng LH, Almeida RP, Carvalho EM. Evaluation of IFN-gamma and TNF-alpha as immunological markers of clinical outcome in cutaneous leishmaniasis. Rev Soc Bras Med Trop **2002**; 35:7-10.

12. Faria DR, Gollob KJ, Barbosa JJ, et al. Decreased in situ expression of interleukin-10 receptor is correlated with the exacerbated inflammatory and cytotoxic responses observed in mucosal leishmaniasis. Infect Immun **2005**; 73:7853-9.
13. Lopez-Casillas F, Cheifetz S, Doody J, Andres JL, Lane WS, Massague J. Structure and expression of the membrane proteoglycan betaglycan, a component of the TGF-beta receptor system. Cell **1991**; 67:785-95.
14. Blobe GC, Schiemann WP, Pepin MC, et al. Functional roles for the cytoplasmic domain of the type III transforming growth factor beta receptor in regulating transforming growth factor beta signaling. J Biol Chem **2001**; 276:24627-37.
15. Border WA, Noble NA. Transforming growth factor beta in tissue fibrosis. N Engl J Med **1994**; 331:1286-92.
16. Santibanez JF, Quintanilla M, Bernabeu C. TGF-beta/TGF-beta receptor system and its role in physiological and pathological conditions. Clin Sci (Lond) **2011**; 121:233-51.
17. Morgado FN, Schubach A, Rosalino CM, et al. Is the in situ inflammatory reaction an important tool to understand the cellular immune response in American tegumentary leishmaniasis? Br J Dermatol **2008**; 158:50-8.
18. Zhou XD, Xiong MM, Tan FK, Guo XJ, Arnett FC. SPARC, an upstream regulator of connective tissue growth factor in response to transforming growth factor beta stimulation. Arthritis Rheum **2006**; 54:3885-9.
19. Bergmeier V, Etich J, Pitzler L, et al. Identification of a myofibroblast-specific expression signature in skin wounds. Matrix biology : journal of the International Society for Matrix Biology **2018**; 65:59-74.

Supplementary Information Table 1. Signals of Association at Candidate and Related Loci Previously Reported as Genetic Risk Factors for CL Caused by *L. braziliensis*

Chr	Position (bp)	rsID	P-value	Odds Ratio (95% CI)	Beta (SE)	Allele[1]	Variant Origin	Location	Gene Symbol[2]	Function (Origin of Variant)
1	86264025	rs380654	2.06E-04	0.95 (0.93-0.98)	-0.29 (0.008)	G (C/G)	Global	Intron	COL24A1	Collagen family member 24A1
1	92303128	rs141665520	3.98E-04	1.15 (1.07-1.25)	0.026 (0.007)	C (G/C)	African	intron	TGFBR3	TGF-β receptor 3
1	92306871	rs77138249	3.98E-04	1.15 (1.07-1.25)	0.026 (0.007)	G (T/G)	African	intron	TGFBR3	TGF-β receptor 3
1	103417667	rs114806195	6.22E-04	0.90 (0.85-0.96)	-0.025 (0.007)	T (T/A)	Global	intron	COL11A1	Collagen family member 11A1
1	103418632	rs111420962	6.22E-04	0.90 (0.85-0.96)	-0.025 (0.007)	G (A/G)	Global	intron	COL11A1	Collagen family member 11A1
1	154428283	rs12133641	7.14E-04	0.96 (0.94-0.98)	-0.027 (0.008)	A (G/A)	Global	intron	IL6R	Interleukin 6 receptor
4	146477459	rs76796874	7.49E-04	1.15 (1.06-1.24)	0.026 (0.008)	C (G/C)	African	intron	SMAD1	R-SMAD[3]
13	37445256	rs8001427	0.004	1.13 (1.04-1.23)	0.022 (0.008)	G (A/G)	African	Intron	SMAD9	R-SMAD[3]
15	67079073	rs59361088	0.001	0.86 (0.79-0.94)	-0.024 (0.007)	G (C/G)	African	downstream	SMAD6	I-SMAD[3]
15	67364991	rs4776880	0.009	0.96 (0.93-0.99)	-0.021 (0.008)	A(G/A)	Global	intron	SMAD3	R-SMAD[3]
18	45390603	rs115582038	1.47E-04	1.19 (1.09-1.30)	0.028 (0.007)	C (G/C)	African	intron	SMAD2	R-SMAD[3]
18	45393852	rs75753347	1.47E-04	1.19 (1.09-1.30)	0.028 (0.007)	G (A/G)	African	intron	SMAD2	R-SMAD[3]
18	46454258	rs9956511	0.005	0.86 (0.77-0.95)	-0.021 (0.008)	T (A/T)	African	intron	SMAD7	I-SMAD[3]
18	48562852	rs78801230	6.02E-04	1.15 (1.06-1.25)	0.026 (0.008)	T (A/T)	European	intron	SMAD4	Co-SMAD[3]
18	48604010	rs1241993461	1.90E-04	1.17 (1.08-1.27)	0.028 (0.008)	A (AT/A)	Rare	intron	SMAD4	Co-SMAD[3]
21	34654499	rs2247177	0.003	0.97 (0.95-0.99)	-0.023 (0.008)	G (A/G)	Global	intron	IL10RB	Interleukin 10 receptor B

NOTE [1]Associated allele (ancestral/minor) for risk or protection as indicated by the odds ratio. [2]Data for two variants provided when the top two hits were in linkage disequilibrium. [3]SMADs are structurally similar proteins that are the main signal transducers for receptors of the transforming growth factor beta (TGF-β) superfamily. The abbreviation derives from homologies to the *Caenorhabditis elegans* SMA ("small" worm phenotype) and Drosophila MAD ("Mothers Against Decapentaplegic") family of genes. R-SMAD = receptor-regulated SMAD; Co-SMAD = common partner SMAD; I-SMAD = inhibitory SMAD.

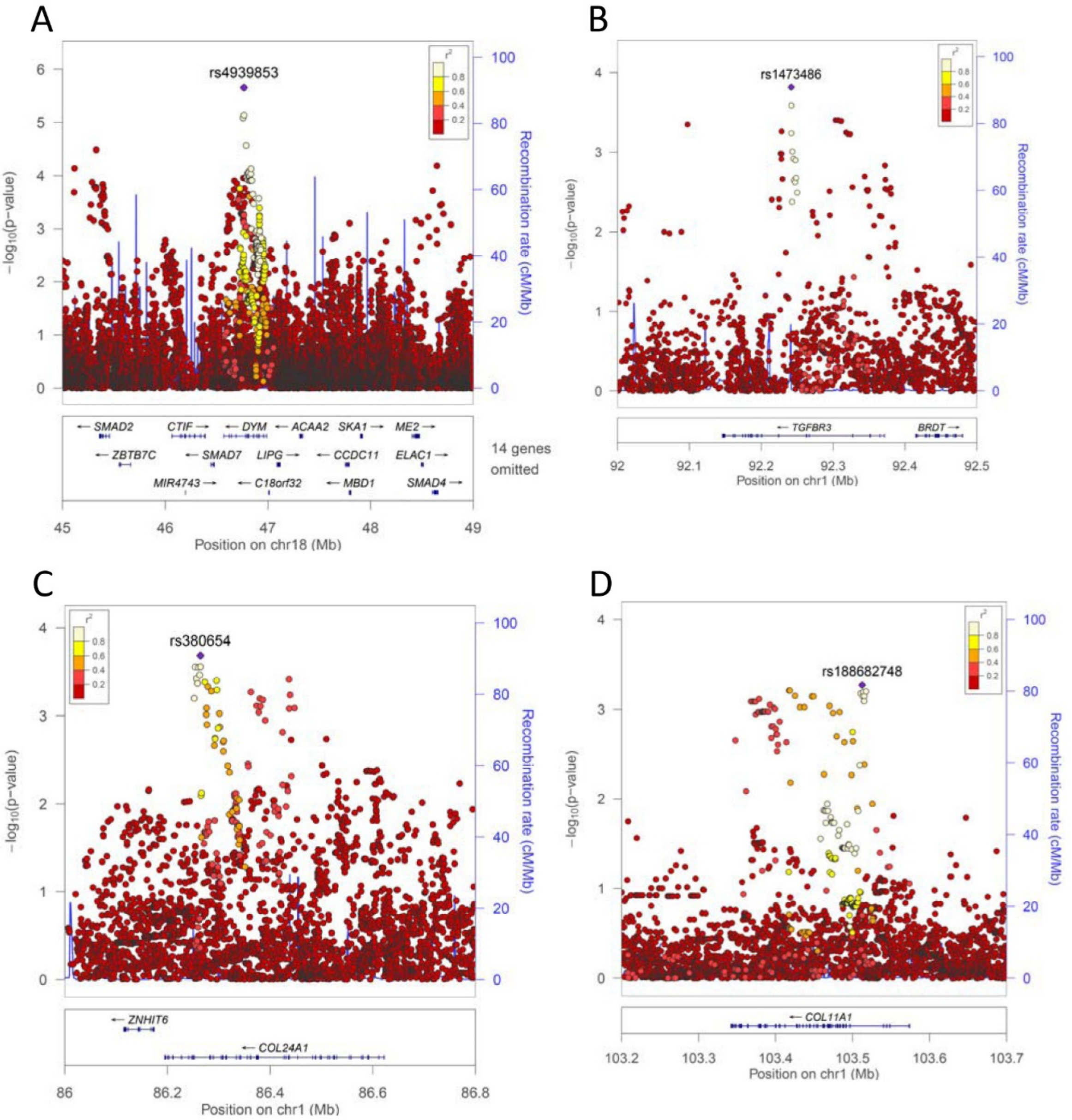

Supplementary Information Figure 1. LocusZoom plot of single-nucleotide variant (SNV) associations with CL across regions of chromosomes containing genes previously associated with, or functionally related to, CL in candidate genes studies. The –log10 *P* values (left y-axis) are shown in the top section of the plot. Dots representing individual SNVs are color coded (see key) based on their population-specific linkage disequilibrium r2 with the top SNV (annotated by rs ID) in the region. The right Y-axis is for recombination rate (blue line), based on HapMap data. The bottom section of each plot shows the positions of genes across the region. The genes of interest include (A) *SMAD2*, *SMAD7* and *SMAD4* (for clarity, 14 genes are omitted from the bottom section of this plot; association at *DYM* is listed in Supplementary Table S3); (B) *TGFBR*3; (C) *COL24A1*; and (D) *COL11A1*.
